# Variations in the Strength of Association between Food Neophobia and Food and Beverage Acceptability: A Data-Driven Exploratory Study of an Arousal Hypothesis

**DOI:** 10.3390/nu13103657

**Published:** 2021-10-19

**Authors:** Sara R. Jaeger, Sok L. Chheang, John Prescott

**Affiliations:** 1 The New Zealand Institute for Plant & Food Research Limited, Mt Albert Research Centre, Auckland 1003, New Zealand; Sara.Jaeger@plantandfood.co.nz (S.R.J.); sok.chheang@plantandfood.co.nz (S.L.C.); 2 TasteMatters Research & Consulting, Sydney, NSW 1230, Australia; 3 Department DAGRI, University of Florence, 50144 Florence, Italy

**Keywords:** neophobia, arousal, liking, consumer research, product research, adults

## Abstract

The negative impact of food neophobia (FN) on food and beverage (F&B) liking extends beyond foods and beverages that are novel. In addition, F&Bs that are high in flavour intensity, perceived as dangerous, or have connections to other cultures are likely to elicit rejection by those high in FN. Each of these factors have been established as producing increased arousal, potentially to an unpleasant degree. The aim of this study was to explore the hypothesis that increased arousal underlies all causes of rejection due to FN. To do this, we analysed and interpreted existing data based on online surveys that measured FN and liking for a broad range of F&B names from 8906 adult consumers in the USA, United Kingdom, Australia, Germany and Denmark. Negative associations between FN and liking of varying strengths were evident for 90% of the F&Bs. Consistent with the arousal hypothesis, F&Bs (a) with high flavour intensity, whether produced by chilli, other spices, or flavours, (b) from other cultures, (c) often perceived as dangerous, or (d) that were novel or had novel ingredients showed the strongest negative relationships between FN and liking. Conversely, F&Bs whose liking scores were only very weakly related to FN had low arousal characteristics: high familiarity, sweetness, mild flavours, strong connections to national food cultures, or some combination of these factors. Since this study was exploratory and conducted on existing data, there was no direct measure of arousal, but this is recommended for future, stronger tests of this arousal hypothesis.

## 1. Introduction

### 1.1. Food Neophobia

Typically defined as a relative reluctance to consume unfamiliar foods or beverages, food neophobia (FN) is recognised as an adaptive trait of omnivores, including humans. In children, a sensitive period beginning towards the end of the second year during which new foods are frequently rejected has been identified [1]. This period is generally considered to be a developmental stage that limits ingestion of unfamiliar, and therefore potentially dangerous, items that might be mistaken for food. FN in children has a significant impact on diet by reducing the number and variety of foods that are tried, as well as the range of foods that are preferred [2,3,4]. 

In adults, FN exists as a continuous trait on which the population is distributed. Based on scores on the Food Neophobia Scale (FNS) [5], the distribution ranges from 10 to 70—from neophilic to highly neophobic—with a positive skew, such that the bulk of the population is usually classified as having low to medium FN [6,7]. Nevertheless, some estimates from population samples in the USA based on a median split of FNS scores suggest that 40–45% of individuals are relatively high in FN [8]. As with children, high FN in adults is associated with reduced dietary variety and more disliked foods [9], reduced intention to try new foods [2], and negative attitudes towards foods originating from other cultures [10].

Although FN is defined, and prima facie measured, as a response to food novelty, there is evidence that high scores on the FNS also reflect rejection of, or lower preference for, foods that vary along dimensions other than familiarity. Thus, although food novelty is an issue for both children and adults high in FN [11], those with higher FN also give lower liking ratings to, and are less likely to consume or even try, many familiar foods [2,6,9,12,13,14,15,16,17]. They also show a reluctance to re-try foods that they have already tasted [15].

High FN has also been linked to differential responses to different types of foods, whether familiar or not. Thus, novel foods of animal origin (meat, seafood, eggs, dairy) tend to generate more negative responses than do novel vegetables, fruits or grains [18]. However, more recent research on large samples (>1000 people) divided according to FN has shown that all food types, novel or familiar, tend to be less acceptable in high FN individuals [6]. Moreover, this was true even for common, everyday consumables including meats, fruits and vegetables. In addition, increasing FN was associated with increasing numbers of disliked foods across all categories, although there was some evidence that seafood was most strongly disliked as FN increased. 

The most frequent explanation of FN is based on the idea, at least in children, that avoidance of unknown foods reduces the risk of consuming potential toxins. It is therefore seen as an evolutionary adaptation in response to the *Omnivore’s Dilemma* [19], an interpretation supported by the large genetic component to FN [20]. While this may be true in children, in the sense that novelty appears to be crucial, these findings suggest that initial responses to food novelty may become more generalised to a broader range of foods in adults, or that food novelty is not the only source of neophobic responses. What has not been determined is whether there is a common denominator, including, but not limited to, novelty of the foods and beverages that adults high in FN tend to reject or find less acceptable than those lower in FN.

### 1.2. Food Neophobia and Arousal

One potential defining feature of responses to F&Bs in those who are high in FN is that they elicit unpleasant levels of arousal. In the psychological literature, arousal refers to a dimension that includes a complex of internal feeling states, autonomic activation and focussed attention [21,22]. When arousal is high, as in emotional states such as fear, the psychological and physiological reactions can be seen as responses to the perception of external threats. Berlyne [23] described the hedonic implications of arousal produced by sensory stimuli in terms of an inverted U-shape such that both low and high arousal were associated with low hedonic value, while hedonic maxima were reached at moderate levels of arousal. Key to this relationship were both the novelty and complexity of the stimuli. Thus, hedonic responses start off low—essentially boredom—for simple, familiar stimuli, rising to peak interest and enjoyment when complexity or novelty are moderate. Subsequent decreases in hedonic evaluations for highly complex and/or very unfamiliar stimuli suggest that these stimuli are the source of unpleasantly high arousal.

### 1.3. Sources of Arousal in Foods

Novelty per se is therefore a source of high arousal and there is evidence that this is due to the fact that unfamiliar foods are perceived as more potentially dangerous than are familiar foods [24]. However, high FN individuals actually appear to be hyper-reactive to foods, irrespective of their novelty. This is seen in increases in physiological indices of arousal when viewing pictures of foods [25] or touching actual foods [26], and decreased sniffing of food odours [27] all independent of food familiarity. In addition to stimulus novelty, high arousal is also a function of complexity and intensity in sensory stimuli [23,28,29]. Thus, high FN is associated with reduced liking for, and choice of, foods that are intensely flavoured, e.g., those foods that are bitter, astringent or high in pungency [12,13]. This has been interpreted as due to higher arousal in response to inherent warning signals that foods may be dangerous to consume. Hence, foods that are strongly bitter induce stronger responses in physiological measures that reflect arousal [29]. The same effect on food acceptability appears to occur when foods are higher in complexity, such that bland foods were overall more acceptable to high FN individuals than were complex flavoured foods, while neophiliacs showed no such difference [30].

The potential role of high arousal in FN has been supported by experimental studies that have manipulated arousal and examined the impact on selection of novel foods. Pliner et al. [24] induced fear by informing participants that they would be giving an impromptu speech to other students, finding that this group chose fewer novel foods than a low fear group, at least when hunger was low. Manipulating arousal using a video game, Pliner and Melo [31] found that low arousal participants ate significantly more novel foods than when the game produced high arousal. Those who were high in the trait of seeking out new sensations—sensation seeking [32]—were much more likely to choose novel foods when arousal was low, but when arousal was high, the effect of sensation seeking on novel food choice was minimal.

### 1.4. Food Neophobia and Sensory Sensitivity

The relationship between FN and arousal may actually be a reflection of a more general heightened responsiveness to stimuli by those high in FN. This is evident in the close relationship between FN and more general (that is, not food-related) stimulus neophobia [2,5,33]. In children, high levels of negative emotional responses to novelty per se may be a risk factor for developing high FN [34]. In addition, high FN is associated with general trait anxiety [5] and lower sensation seeking [5,31,35]. FN also tends to cluster with other arousal-related personality traits including disgust sensitivity and sensitivity to punishment [36,37] when the impact of pungency on food choices is studied. There is even some evidence that FN is linked to aspects of social anxiety, in that high FN individuals seem less open to other cultures in general, not just in terms of their cuisines [38].

Indeed, both anxiety and a broad sensory sensitivity have been shown to be associated with selective/picky eating in children [39], and such sensitivity also appears to link anxiety and FN in young adults [40]. One consequence of such sensitivity may be attentional biases towards novelty in foods, which in children is evident, generally when presented with unfamiliar fruits and vegetables, but which is much stronger in those with high FN [41]. Such attentional biases towards threatening stimuli are a characteristic of anxiety disorders in adults [42].

### 1.5. Research Aims and Approach

Based on these earlier findings, the present research aimed to explore if relationships between FNS scores and F&B acceptability ratings in an existing data set drawn from multiple countries was consistent with arousal as a unifying explanation for degree of neophobic response. We hypothesised that F&Bs with pre-determined characteristics that potentially induced arousal would more likely be rejected or found less acceptable by adults higher in FN than by those lower in FN. Although we did not employ a direct measure of the arousal produced by these foods (or rather, their names), there is sufficient evidence, as noted above, that F&B characteristics such as novelty (including foreignness), complexity, perceived dangerousness, and intensity are rejected by those high in FN *and* are associated more generally with increased arousal.

Specifically, we examined responses of consumers measured on the FNS, and from a variety of countries, to names of F&Bs that were selected to vary along multiple dimensions including overall novelty, novel ingredients/unexpected combinations of ingredients in familiar foods, flavour intensity, perceived ‘dangerousness’ and being familiar but polarising. We expected that F&Bs high on one or more of these dimensions would show reduced acceptability in those with high FN and hence be consistent with an explanation that implicated higher arousal. Hence, the study reported here consists of a hypothesis-driven exploration and interpretation of existing data. Eight consumer studies in five Western countries contributed to the research with responses from a total of 8906 adult participants (Table 1). The data were collected in online surveys over a two-year period and involved 219 F&Bs. An important reason to use online surveys in FN research is to help overcome the low participation rates of those high in FN in central location tests [43].

## 2. Materials and Methods

### 2.1. Participants

The participants were members of online panels managed by ISO accredited research providers. Table 1 shows the number of participants in each of the eight studies, their age range and male/female split. See Part 1 of Supplementary Materials for full participant details.

### 2.2. Empirical Approach

#### 2.2.1. Trait Food Neophobia

The 10-item trait Pliner and Hobden Food Neophobia scale (FNS) [5] was used with 7-point Likert scales (1 = ‘disagree strongly’ to 7 = ‘agree strongly’). In Study 1, six of the ten items in Pliner and Hobden [5] were used, selected as those items that Ritchey, Frank, Hursti and Tuorila [44] identified as an appropriate subset for USA following a scale refinement study. In Study 2, 50% of participants used the 10-item FNS and 50% of participants used the 6-item FNS. In all studies, the presentation order of FN scale items was randomised.

#### 2.2.2. Product Names

F&B names were used as stimuli and enabled coverage of a large number of products. This was essential to achieve the research aim, and across the eight studies, a total of 219 names were used (full listing in Part 3 of Supplementary Materials).

While the selection of F&B names in individual studies was partly subject to criteria determined by other research aims, care was taken to ensure diversity across the eight studies in accordance with several guiding principles. Foremost, stimuli with probable low/moderate hedonic appeal to neophobic individuals (e.g., dishes from other cultures, seafood/shellfish, offal, strong/spicy flavours) were included. There was a range of more/less familiar foods (e.g., ‘instant noodles’ vs. ‘eggs Benedict’ (USA); ’rice and milk porridge’ vs. ‘cheese fondue’ (Denmark)). Foods and beverages were both represented, but the latter more infrequently, fitting with less diversity in this category. There was variation to capture breakfast, lunch and dinner F&Bs (e.g., ‘porridge/hot oatmeal’, ‘ham and cheese sandwich’, ‘prawn risotto’), as well as snack and sweet F&Bs (e.g., ‘mixed raw nuts’, ‘lemon mousse tart’), hot and cold F&Bs (e.g., ‘hot coffee’, ‘frozen yoghurt’), fruits and vegetables (e.g., ‘apple’, ‘mixed green salad’) and meat and vegetarian dishes (e.g., ‘spaghetti Bolognaise’, ‘vegetable and bean hot pot’). Some stimuli contained novel ingredients (e.g., ‘granola bar with insect flour’) or combined known ingredients in untraditional ways (e.g., ‘zucchini brownie’, ‘beef and beetroot sausages’). Some stimuli were only slightly different (e.g., ‘apple and kale juice’ vs. ‘apple, orange and kale juice’; ‘vegetarian pizza’ vs. ‘seafood pizza’, ‘tuna steak’ vs. ‘tuna pasta’). The F&B names were developed by the authors and revised by sensory and consumer professionals with appropriate knowledge of eating and drinking habits in the different countries.

Consumers’ liking responses were obtained on 9-point fully-labelled hedonic scale (1 = ‘dislike extremely’ to 9 = ‘like extremely’) [45]. The F&B names were presented according to a randomisation design. 

#### 2.2.3. Data Collection and Previous Use

Participants completed the survey in a location of their choosing. Stated liking data were always collected before FN. Demographic and socio-economic responses were obtained last. In all studies, data additional to those reported here were obtained, but not reported due to lack of relevance for the research aim.

The data for Studies 1 and 4 were previously used by Jaeger, Roigard, Hunter and Worch [46]. The data for Study 4 were also used by Jaeger, Roigard et al. [47], while those for Study 8 were used by Jaeger, Roigard, Ryan et al. [43]. However, these data have not previously been analysed collectively with a view to exploring arousal as a unifying explanation for neophobic responses.

### 2.3. Data Analysis

The same procedures were used in all studies, with analyses performed in XLSTAT v.2020.3.1 (Addinsoft, 2020) using a 5% significance level. The data analysis strategy was borrowed from approaches used in several recent studies on related topics [43,46,48].

#### 2.3.1. Food Neophobia

Following standard practice, summed scores (following reverse coding of required items) were calculated across all scale items, and in all studies, a continuous distribution of FN scores across the theoretically possible range (10 to 70) were observed. In Studies 1 and 2, summed FN scores from the 6-item FNS were scaled to reflect the comparative values if 10 scale items had been used rather than 6 scale items (1.667 multiplier) (A comparison of summed scores from the two FNS sub-samples in Study 2 found these to have similar means based on the 6-item FNS (p = 0.10) and supported a joint analysis). In all studies, values for Cronbach’s alpha exceed the typical 0.7 threshold [49] to indicate acceptable internal consistency of the FN scale.

#### 2.3.2. Regression and Discretisation

For each F&B item, the relationship between FN and degree of liking/disliking was modelled using linear regression. Drawing directly on Jaeger et al. [43,46], the regressions were performed on mean values for liking calculated for each FN scale point (FN = 10, FN = 11, FN = 12, etc.). These mean liking scores were based on different numbers of consumers due to the shape of the FN distribution (Part 4 of Supplementary Materials). Goodness of fit (R^2^), regression coefficients (b) (with 95% confidence intervals) and intercepts (a) were retained for further evaluation. Of the 219 regression models, 199 (91%) were significant at p < 0.05 (Part 3 of Supplementary Materials lists those F&B items where significant models were not established).

The 199 F&Bs where a significant relationship was found between liking and FN were input to further analysis, seeking a meaningful reduction in this large number of individual F&Bs that would enable hypothesis exploration. Accordingly, a discretisation procedure was applied to the distribution of the 199 regression coefficients to obtain an empirical separation of the F&Bs into different groups based on the extent to which FN influenced average liking. The number of groups was determined by evaluating solutions from 4 to 12 and considering the relative frequency of F&B names in each group and its interpretability. It was decided to retain six groups since n ≥ 7 only resulted in ongoing sub-division of the two groups with least F&B items, and a more fine-grained distinction between F&B items where the regression coefficients were negative but very close to zero.

To facilitate the presentation of results, and, in turn, hypothesis exploration, names were assigned to the six groups of F&Bs. These names were descriptive labels that differentiated the groups based on the direction and relative magnitude of the effect of FN on liking, as follows: “negative and very high” effect (18 F&B names), “negative and high” effect (54 F&B names), “negative and medium” effect (64 F&B names), “negative and low effect” (34 F&B names), “negative and very low effect” (27 F&B names) and “positive and very low effect” (2 F&B names) (Part 5 of Supplementary Materials provides a descriptive summary of the six groups).

## 3. Results

The present research was a hypothesis-driven exploration of arousal as a unifying explanation for degree of neophobic response, and we expected that F&Bs with arousal-inducing characteristics would be less acceptable to individuals higher in FN. The empirical results were interpreted against this background, focussing on groups of F&B items where F&B liking decreased with FN.

### 3.1. Characteristics of F&B Items in the Groups Where Strength of the Negative FN-Liking Relationship Differs

By group, Table 2 lists the 197 F&B names with negative regression coefficients in ascending order. They ranged from −0.132 (‘Thai green chicken curry,’ UK) to −0.002 (‘Danish pastry,’ DK), meaning that a 10-point increase in FN equates to a decrease in liking of, respectively, 1.3 and 0.02 scale points on the 9-point hedonic scale. This highlights the substantial effect of product category/type on the impact exerted by FN on liking (Part 6 of Supplementary Materials shows this visually).

To explore the arousal hypothesis as an explanation of the degree of neophobic response, the five groups of F&Bs with negative regression coefficients are considered below, focussing on those characteristics that are shared among F&B items in one or more group, but absent in other groups (Table 3). These categories of F&B characteristics were derived by considering the items in the “very high” group and their unique characteristics. Progressing to the “high” group, new categories of F&B characteristics were added to describe the F&B items in this group not already captured by existing categories. The process continued in this manner until the “very low” group and any new categories needed to capture specific characteristics of its F&Bs. The resulting categories of F&B characteristics were diverse spanning aspects linked to sensory properties, ingredient combinations, product category, familiarity, cultural origins, etc. Multiple categories could apply to individual F&B names.

The process of sequentially deriving the categories of F&B characteristics—progressing from the group where F&B items evoked the strongest neophobic responses to the group where F&B items evoked the smallest neophobic response—was deliberate and aligned with the hypothesis of arousal as unifying explanation for F&B dislike and rejection. Thus, we expected to uncover an ordering of the categories of F&B characteristics from more to less arousal-inducing.

#### 3.1.1. “Very High” Negative Effect of FN on F&B Liking

The 18 F&B items in the group labelled as “very high” in reference to the negative effect of FN on liking shared one or more of the characteristics: *From other cultures* (curry, korma, Thai, kebab, enchilada), *Chilli/Spicy* (‘chilli con carne,’ ‘chilli chicken stir-fry,’ ‘spicy lamb meatballs’), and *Shellfish/Sushi* (‘sushi,’ ‘prawn risotto’). F&B items with explicit mention of chilli and spicy were found in both this cluster (i.e., “very high”) and the “high” group, but items that explicitly or indirectly referenced “curry” were only found in this group (‘Thai green chicken curry,’ ‘chicken korma’), and notably, this extended to ‘mild Indian curry (vegetarian)’ (UK). ‘Sushi’ featured in four studies and three countries (US, AU, DK), and the 95% confidence intervals around the regression coefficients overlapped, suggesting that the same highly negative effect of FN was country independent. There were no beverages in the “very high” group.

#### 3.1.2. “High” Negative Effect of FN on F&B Liking

The categories of F&B characteristics that defined membership of the “very high” group—*From other cultures, Chilli/Spicy* and *Shellfish/Sushi*—were also represented in the “high” cluster (e.g., ‘breakfast burrito’ (US), ‘chicken and rice salad with spicy mayonnaise’ (DE) ‘steamed mussels’ (US)), although there were only three F&B items in this cluster that explicitly mentioned chilli or spicy/hot, and these were all placed in the top quartile of the group according to the value of the regression coefficient. 

The number of F&B items in the “high” group—54—was much larger than the “very high” group and considerably more diverse, leading to additional categories of F&B characteristics (Table 3). The first of these—*Strong flavour*—referred to strong flavours other than chilli/spicy in accordance with the “hierarchical” coding process described above, and F&B items representing this category were ‘strong mustard’ (AU), ‘blue-vein cheese’ (AU), ‘smoked cheese’ (AU) and ‘pasta with sundried tomato and garlic meat sauce’ (DE).

The category named *Dish with reduced familiarity* were introduced in this group to encompass F&B items with reduced familiarity that were not already covered by existing categories including *From other cultures*. Exemplar F&B items were ‘cheese fondue’ (DK) and ‘three cheese and chorizo omelette’ (US). Additional new categories relating to higher/lower familiarity were: *Familiar F&B from novel ingredients* (‘oat milk with cocoa flavour’ (US), ‘vegan meat balls made with soy protein’ (US), ‘granola bar with coconut and chia seeds’ (US)), *Familiar F&B but often disliked* (e.g., ‘fried mushrooms’ (US), ‘wholemeal pasta salad with chicken’ (DE), ‘root vegetable stew’ (US)), and *Familiar F&B with unusual ingredients/aspect* (e.g., ‘apple, orange and kale juice’ (US), ‘toasted rye bread with fried eggs and avocado’ (DK) and ‘roasted nuts’ (DK)).

The “high” group included nine items containing shellfish or fish, and six of these had regression coefficients that placed them in the top half of the group (i.e., closer to the “very high” than to the “medium” group): ‘steamed mussels’ (US), ‘seafood pizza’ (US), ‘fried oysters’ (US), ‘shrimp taco’ (US), ‘stuffed crust pizza with cheese, tomato and shrimp’ (US) and ‘tuna steak’ (AU, UK)). In comparison, the remaining three items (‘baked salmon’ (US), ‘tuna pasta’ (UK) and ‘seafood chowder’ (US)) were more familiar and/or part of dishes where the fish/seafood flavour was a less dominant component. *Beans/Legumes* (e.g., ‘refried beans’ (US), ‘kidney bean salad’ (US), ‘salsa black bean burger’ (DE)) and *Unusual meat/Offal* (e.g., ‘rabbit ragu’ (AU)) were the final two categories of F&B characteristics created to describe the items in this group.

#### 3.1.3. “Medium” Negative Effect of FN on F&B Liking

The largest group of F&B items (n = 64) was extremely diverse (Table 3) and drew further attention to the fact that the negative effect of FN on liking is pervasive rather than being limited to strictly novel foods and beverages. F&B items which explicitly mentioned chilli were absent, and only one item mentioned spicy (‘spicy red cabbage’ (DE)). Connotations to other cultures were most obvious in ‘breakfast burrito’ (US), but also implied in ‘pickled herring’ (AU) and ‘skyr with muesli’ (DK) (skyr is an Icelandic variant of yoghurt). Items with *Strong Flavour* (other than chilli/spicy) such as ‘pickled beet and onion salad’ (US) and ‘tossed green salad with red onions’ (US), were infrequent also. These findings combined with the absence of *Shellfish/Sushi* items in the “medium” group highlighted its difference relative to the “very high” and “high” groups, and, more generally, how the negative effect of FN, although weaker, was still systematically linked to various categories of F&B characteristics. 

Distinct from the category *Shellfish/Sushi*, there were seven F&B items with *Fish* in the “medium” group—‘sardines on toast’ (UK), ‘crackers with salmon pate’ (UK), ‘baked salmon’ (US), ‘tuna steak’ (UK), ‘pickled herring’ (AU), ‘salmon and green salad’ (DK) and ‘fish fingers’ (US). Among the other product specific categories, *Unusual meat/Offal* was represented by: ‘liver pate’ (US), ‘tripe and onion’ (US), ‘baked rabbit’ (US) and ‘veal burger’ (US)), and the former two items, such as ‘pickled herring’ probably placed in this lower-than-expected group due to widespread disliking (Table 3). New product-focussed categories of F&B characteristics identified in the “medium” group were *Vegetables/Salads*, represented by ‘mixed green salad’ (US, UK, AU), ‘Caesar salad’ (AU), ‘mixed grilled vegetables’ (DK), ‘raw snack vegetables’ (AU)) and *Soup*. The latter appeared as a category that was largely specific to the “medium” group: ‘lentil and beet soup’ (US), ‘cream of mushroom soup’ (US), ‘corn chowder’ (US), ‘potato and lentil soup’ (DE) and ‘ham and potato soup’ (DE). However, there was a partial overlap with *Beans/Legumes*, which likely exerted a notable negative effect, based on the results for the “high” group. 

The categories of F&Bs’ characteristics relating to familiarity/novelty were similar to those identified in the “high” group. F&B items representing the category *Dish with reduced familiarity* were: ‘stuffed bread with cheese and herbs’ (DE), ‘cheese fondue’ (DK), ‘lamb stew’ (US) and ‘savoury mince’ (UK). Extending from there: *Familiar F&B from novel ingredients* (e.g., ‘burger with patty from 100% plant-based meat substitute’ (AU), ‘dairy-free yoghurt’ (UK), ‘granola bar with insect flour’ (US)), *Familiar F&B but often disliked* (e.g., ‘vegetable juice’ (DK), ‘herbal tea’ (DK), ‘stewed apples’ (DK), ‘beer’ (DK), ‘hot coffee’ (US), ‘Brussel sprouts’ (AU), ‘mixed nuts with dried fruits’ (DK)) and *Familiar F&B with unusual ingredients/aspects* (e.g., ‘zucchini brownie’ (US), ‘beef and beetroot sausages’ (UK), ‘apple and kale juice’ (AU), ‘ham and tomato muffin’ (AU), ‘vegetarian meat loaf’ (DE), ‘iced coffee’ (AU), spinach and tomato omelette’ (DK) and ‘stuffed bread with cheese and herbs’ (DE)). Familiarity for other items was reduced due to inclusion of a less known ingredient (e.g., ‘bun (Focaccia bread) with turkey, salad and dressing’ (DK) and ‘skyr (Icelandic yoghurt) with muesli’ (DK)). 

A number of F&B items—‘sparkling water’ (DK), ‘club soda’ (US), ‘yoghurt’ (AU), ‘chicken casserole’ (DE) and ‘egg mayonnaise sandwich’ (AU)—did not appear to fit within the categories of F&B characteristics that defined membership of the “medium” group. However, new categories formed in the interpretative process for the “low” cluster did appear to represent several of these items, and it was unclear why the negative effect of FN was not lower. 

#### 3.1.4. “Low” Negative Effect of FN on F&B Liking

In this group (n = 34), the absence of several of the categories of F&B characteristics observed in the three previous groups was noted (Table 3). There were no F&B items which directly identified them as being from other cultures, and there was only a single instance of reference to chilly/spicy: ‘chilli cheese dog’ which is a relatively common food in the US. Neither were there any F&B items representing the categories *Strong Flavour*, *Shellfish/Sushi* and *Unusual meat/Offal*. There were three *Fish* items, although ‘herring fillet in tomato sauce’ (DE) and ‘fish cake on bread’ (DK) are highly familiar and widely consumed, which is also true of ‘tuna salad sandwich’ (US). In the latter, the fish taste is also likely masked by other ingredients such as mayonnaise, making it more widely acceptable. The category capturing *Familiar F&B with novel ingredients* was missing from the “low” group, as were items containing *Beans/Legumes*. There was only a single F&B item representing the *Vegetable/Salad* category—‘raw vegetables: tomato, cucumber, cauliflower, capsicum’ (DK).

Items representing the categories of F&B characteristics named *Familiar F&B but often disliked* and *Familiar F&B with unusual ingredient/aspect* were well represented in the “low” group. The former included: ‘cheese and vegemite sandwich’ (AU), ‘dark chocolate’ (AU), ‘wholemeal bread with jam’ (DK), ‘peanut butter sandwich’ (AU), ‘lamb chops’ (US), ‘quiche with leek and bacon’ (DK) and ‘porridge/hot oatmeal’ (AU). Representatives of the latter category were: ‘white bread roll with ham and cheese’ (DK), ‘burger with patty from ground beef and vegetable blend (50:50)’ (US), ‘strawberry flavoured milk (from cows)’ (US) and ‘pork and potato sausages’ (UK).

Additional categories of F&B characteristics emerged clearly in the “low” group: *Familiar hot meals with meat* and *Familiar and grain-based (cold)*. The former included: ‘spaghetti Bolognaise’ (DE), ‘chicken casserole’ (UK), ‘All-American beef stew’ (US) and ‘beef rissole and potato salad’ (DK). The latter included: ‘bread and cheese’ (DE), ‘muesli with milk’ (DK), ‘ham and cheese muffin’ (AU), ‘rye bread with sliced meat’ (DK) and ‘rye bread with cheese’ (DK). Several popular non-alcoholic cold beverages were also found in this cluster: ‘sparkling water’ (AU), ‘water’ (AU) and ‘strawberry and banana smoothie’ (US).

The unexpected placement of ‘fried liver’ (US) and ‘pickled pigs’ feet’ (US) in the “low” group can be attributed to widespread dislike, irrespective of degree of FN. For example, the estimated average liking for ‘pickled pigs’ feet’ was lower than 3 on the 9-point scale even among the most neophilic participants. A similar explanation applies to ‘stewed prunes’ (US). 

#### 3.1.5. “Very Low” Negative Effect of FN on F&B Liking

Fitting the earlier patterns, the 27 F&B items in the group where FN exerted a “very low” negative effect on F&B liking were very diverse. The findings from the “low” group in relation to the absence of certain categories of F&B characteristics largely replicated: from other cultures, chilli/spicy, strong flavour, shellfish/sushi, fish, unusual meat/offal, novel ingredients, beans/legumes, vegetables/salad and familiar with unusual ingredients/aspects. The only exceptions were ‘garlic bread’ (AU) and ‘warm liver pate with mushrooms’ (DK). For ‘garlic bread’, it seems plausible that Australian participants based on commercial offerings did not consider this to have *Strong flavour*. In Denmark, warm liver pate with mushroom is a highly popular topping on open sandwiches (smørrebrød) and common on weekend lunches. 

Items fitting into categories of F&B characteristics also observed in the “low” group were: *Familiar hot meals with meat* (e.g., spaghetti Bolognaise’ (AU, DK), ‘beef lasagna’ (US), ‘meat loaf’ (DE) and ‘chicken sandwich’ (US; aka, chicken burger)), *Familiar and grain-based (cold)* (e.g., ‘cereal/muesli’ (US)), ‘ham and cheese sandwich’ (AU) and *Familiar F&B but often disliked* (e.g., ‘coffee’ (DK) and ‘soft boiled egg with bread’ (DK)). 

Categories of F&B characteristics not apparent in the previous groups were: *Familiar desserts/cakes* (‘lemon mousse tart’ (AU), ‘blueberry muffin’ (AU) and ‘Danish pastry’ (DK)), *Fruit* (‘apple’ (AU), ‘banana’ (AU)) and *Mild flavour* (‘mild cheese’ (AU), ‘white rice’ (AU) and ‘cold sliced meats’ (AU)).

## 4. Discussion

Food neophobia, with its negative consequences for food enjoyment and dietary quality [6,9,50], has attracted much scholarly interest. Yet, even though it has become increasingly apparent that novelty is neither necessary nor sufficient to explain the food rejections of those high in FN, an alternative common denominator of the F&B characteristics that adults high in FN find less acceptable has not been established. In the current data set, liking for the vast majority of the 219 F&B items, across a variety of countries, was negatively associated with FN, thus supporting conclusions from previous studies that FN in adults is not related only to novelty but also encompasses foods that are familiar [6]. Lower preferences for, and consumption frequency of, common food items therefore require a consideration of what factors other than novelty might also be involved. In the present research, we explored if there was evidence that arousal—specifically, unpleasantly high arousal—could be a likely candidate.

This hypothesis was developed taking into account evidence that foods per se are generally more arousing for those high in FN. This is evident in measures of arousal when viewing pictures of foods [25] or touching foods [26], as well as a general wariness when sniffing food odours [27]. It is possible, therefore, that foods and eating are more frequently associated with anxiety for those high in FN, perhaps due to fear that they may encounter an unfamiliar or unpleasant taste [24]. Consistent with this notion, experiencing foods tends to reduce the impact of FN on preferences whether the food is initially unfamiliar or not [17,24].

### 4.1. Arousal and Neophobic Responses

Although the original purpose of collecting these data was not to test a hypothesis about the effects of F&B characteristics on arousal, the results are consistent with our post hoc hypothesis that arousal may be a substrate for F&B disliking and rejection. If our hypothesis had no explanatory value for neophobia-based food rejections, then we would expect that liking for *familiar* F&Bs with intense flavours or links to other cultures, for example, would show no relationship with FN. Clearly, this was not the case.

The F&B items in the group where the negative regression coefficients between liking and FN were “very high”—and to a lesser extent the F&B items in the ”high” group—implicated all of the expected arousal-inducing categories of F&B characteristics: flavour intensity whether produced by chilli, other spices or flavours, foods from other cultures (even if familiar), and the novelty of a dish or its ingredients. This is consistent with some general categories of arousal elicitation—particularly, intensity and novelty—that have been previously described [12,13,23,28,29]. Many items within these overlapping categories of F&B characteristics may also elicit arousal due to their perceived complexity, although we have no way of estimating this from the present data. Some seafood items were also present in these groups of high negative effect of FN on liking. This should be viewed in the light of evidence that seafood is commonly considered to contain inherent risks (e.g., contaminants) in its consumption [51,52,53,54], and is therefore considered dangerous, and thus arousal-inducing, relative to other common F&Bs.

Of relevance to the idea of arousal as a unifying factor was the fact that these findings (Table 2), especially for the “very high” group, were relatively uniform across different countries. It has previously been noted that preferences for relatively bland foods—bread, rice, potatoes—tend to be unaffected by FN [6]. This was replicated here, a finding that is also consistent with our arousal hypothesis. Those F&Bs whose liking scores were only very weakly related to FN possessed characteristics not expected to induce arousal: high familiarity, sweetness, mild flavours, strong connections to national food cultures, or some combination of these factors. Additionally, it has been suggested [55] that food with high energy content—often sweet or high in fat, such as more common in the “very low” group—may be less likely to be perceived as unsafe and hence be limited in their ability to elicit neophobic responses because of their potential survival value.

### 4.2. F&B Characteristics and Neophobic Responses

While there was evidence to support arousal as a unifying explanation for F&B rejection and dislike by high FN individuals, this explanation does not necessarily account for all the observed results. In between the extremes of the “very high” and “very low” groups, seemingly without an obvious linkage to arousal, a broad range of categories of F&B characteristics were associated with some degree of neophobic response (Table 2). This could suggest that F&B characteristics other than those addressed by the extant literature, chiefly novelty, complexity and intensity [2,13,30], are at play, and/or that increased arousal explains strong neophobic responses, but not neophobic responses of intermediate strength. The F&B items in those groups where the negative effect of FN on liking was only moderate (“medium” and “low”) were also less uniform across cultures, as might be expected if culture-specific reasons for rejection were more influential in these groups. Alternatively, the low arousal associated with simple, familiar and low intensity foods might be a source of boredom in those low-moderate in FN [56] but could represent sought-after characteristics for the high FN individual.

Although the categories of F&B characteristics rather than the individual items herein were the key to addressing the research aim, it is appropriate to comment on the strength of the FN-liking relationship for F&Bs when notably different to expectations. These “discrepancies” might reflect factors other than FN exerting an influence on liking. As one example, the regression coefficient for ‘prawn risotto’ (AU) meant that this item fitted in the “very high” group despite its ingredients not being unfamiliar, especially exotic or strongly flavoured, although there may be unfamiliarity in the sense that it may not be commonly eaten (see also earlier comments regarding seafood). In addition, in the UK, curries are highly familiar dishes that are no longer especially associated with other cultures, and yet ‘mild vegetarian curry (vegetarian)’ and ‘chicken korma’ were both in the “very high” group for this UK sample. However, some people still find curry of any sort too spicy, and this is probably a function of several things including FN, but also sensory sensitivity, as demonstrated by the reported close relationship of sensitivity to perception of pungency and rejection of pungent foods [37]. 

These and other discrepancies could also point to differences between the way familiarity and novelty in F&Bs are operationally defined here, and the way in which these qualities are perceived by consumers. Thus, certain stereotypical associations may be influential with consumers. For example, based on its regression coefficient, ‘chicken fried rice’ (UK) was placed into the “high” group despite not appearing to meet the derived criteria for membership (i.e., no seafood and not intensely flavoured nor novel in its ingredients). However, fried rice is a popular component of many East Asian and Southeast Asian cuisines with origins in China [57], pointing to a likely perception of ‘chicken fried rice’ as exotic, and hence potentially challenging. 

Based on regression coefficients for the relationship between FN and liking, some items were placed in groups of lower strength than was expected. We propose that such weaker relationships could reflect poor acceptability generally, potentially obscuring any effect of degree of FN. For example, ‘pickled herring’ (AU) might have been expected to fit in one of the two “high” groups considering its strong flavour. However, a likely explanation for why it placed in the “medium” group was the low average liking for ‘pickled herring’ (Section 3.1; Table 2). If an item is generally widely disliked, then the potential for FN to exert a large negative effect (i.e., have a large negative regression coefficient) is reduced. For ‘sardines on toast’ (UK) which also placed in the “medium” strength group, a different explanation seemed likely. Considering its strong flavour, placement in the “high” strength group could have been expected, but the long history of eating sardines in the UK (www.foodsofengland.co.uk, accessed on 20 June 2021) may have exerted an influence in terms of high familiarity. 

Considering the inductive process whereby the categories of F&B characteristics (Table 3) were derived and the dependence of these categories on the items included in the research, it is necessary to acknowledge that they may lack interpretative value in relation to the relationship between FN and liking. The category *Soup* conveniently captured a property that several items had in common, but it is not clear how soup connects to neophobic response since the category spanned from ‘seafood chowder’ (US) which was included in the “high” group and ‘broth with vegetables and meatballs’ (DK) which was included in the “very low” group. Another caution regarding the categories of F&B characteristics is that they are not complete in the sense of providing full coverage of major product classes, e.g., [58]; fruit is missing, for example. This was a consequence of low representation among the 219 F&B items, even though fruit preference and consumption is influenced by degree of neophobia [6]. In particular, it is possible to imagine regression coefficients corresponding to a “medium” strength relationship between liking and FN for fruit that have intense flavours and/or are from other cultures (e.g., durian, gooseberries, pomegranate, mangosteen).

An important consideration in interpreting these data is whether factors other than increases in arousal could account for the observed relationships between FN and liking. The most obvious alternative explanation is that the relationship with FN is with the *names* of the F&Bs, which may be unfamiliar to greater or lesser degrees. Certainly, many items in the “very high” group are, in some sense, more “exotic”, especially in contrast to those F&Bs in the “low” groups, which might be considered more mundane. So, are the “very high” F&B items simply more novel/unfamiliar names? This seems unlikely. For example, given the influx of Asian and Indian restaurants throughout the UK and other Western countries in recent decades, dishes such as chilli chicken stir-fry, mild Indian curry or lamb kebabs are all well known. The same is certainly true of sushi in both the US and Australia. It is relevant to this issue that the data collection was conducted mostly on urban consumers in Western countries, where ethnic restaurants have been common for many years.

### 4.3. Limitations and Suggestions for Future Research

A post hoc interpretation of data that has been collected prior to hypothesis formulation is always likely to raise as many questions as it answers. We regard this as a positive in that it can lead to more explicit hypotheses in later studies. Nevertheless, the results of this exploratory and data-driven analysis should be interpreted with several caveats. The most obvious hurdle faced in this analysis is the absence of a formal measure of arousal. As noted earlier, we believe that our interpretation of responses is consistent with high arousal as an explanation for decreased liking for many foods with increasing FN. However, as a strict test of the arousal hypothesis of FN, future studies ought to include such a measure. Data collection via internet survey, as in the present data set, obviously precludes many of the laboratory-based measures of arousal, e.g., physiological monitoring. Possibly the most practical way of assessing arousal would be to collect ratings on emotions, in addition to those of liking, for each food. It is well established that the underlying structure of emotional terms involves valence and arousal as independent dimensions [59]. Measures of specific emotions such as fear, anxiety, alertness, all of which have a high arousal component, may therefore be highly suitable as direct measures [60]. Another possibility would be to use a measure such as the Affect Grid [22] which contains measures of both arousal and valence, in place of ratings of liking.

The F&B items themselves also do not always provide a straightforward interpretation of the source of high arousal, and it is possible that a more systematic selection of F&B items may have helped in attributing the strength of the relationship between FN and liking to specific F&B characteristics. In the group where FN had a “very high” negative impact on liking, it is possible that a given F&B item was rejected due to it being from another culture, or being spicy, or otherwise having a strong flavour, or a combination of all three factors. In future research, a more systematic variation in F&B characteristics based on arousal potential could help to disentangle this interdependency by creating F&B stimuli that a priori are expected to be associated with strong or weak neophobic responses. A structured approach to stimulus development would also allow for testing of the idea that a food that combines several characteristics typically associated with an “intermediate” neophobic response—perhaps a familiar food with multiple unusual ingredients—may fall into the “very high” group.

Our interpretations relied to a large extent on the discretisation process, which created group boundaries along a continuum from “very high” to “very low” negative impact of FN on F&B liking. While arbitrary, the resulting groups of F&B items nonetheless served as an operational means of describing features that allowed us to identify possible reasons for the relative degrees of rejection of individual F&B items. Other approaches to creating groups of F&B items could have been used, including expert judgment and/or input from consumers in each country. Our placement of individual F&B items into Table 3 is also open to discussion and revision. The inductive process where categories of F&B characteristics were developed and associated with different items was partly driven by the F&B items included in the research and if other stimuli had been used, different categories may have arisen. For example, it is possible to imagine categories for national cuisines and had there been a category for *Italian Food*, ‘prawn risotto’ may have fitted there instead of being placed in the *Shellfish/Sushi* category. For completeness, we note that the absolute values of the regression coefficients between FN and liking for individual F&B names should only be directly compared to other studies with caution since the use of written stimuli, online surveys, and analysis based on aggregate liking values for each FNS scale point all may influence the absolute values.

Finally, the research was conducted in five Western countries, but not designed to specifically address cross-cultural differences in FN, and the selection of F&B items was not made with a view to country-to-country comparison. Rather, in keeping with an exploratory research strategy, a large number of different F&B items which varied in their arousal-inducing potential were included, even if these items occurred in a single country only. Future research could address the important issue of cross-cultural differences more systematically using, for example, items that are consumed (or avoided) in many different countries, such as coffee, sushi, beef burger, pizza, cereal/muesli, apples, bread, eggs, potatoes, chocolate cake, water, beer, Brussel sprouts, liver, etc. A particular point of interest would be to establish how the uniformity of strong neophobic responses in the “very high” cluster evolves in different countries, and how the F&B items become more diverse as the association between FN and food liking weakens.

## 5. Conclusions

The present large data set provides a highly detailed, cross-cultural view of neophobic response to a variety of F&Bs that varied in multiple ways. We hypothesised that food characteristics that potentially induced arousal would be more likely to be rejected as FN increased, and that arousal may act as a unifying explanation for degree of neophobic response. The empirical evidence, consistent with this notion, identified strong negative effects for F&Bs with high flavour intensity whether produced by chilli, other spices or flavours, foods from other cultures, and the novelty of a dish or its ingredients. Additionally consistent with the arousal hypothesis was the finding that F&Bs whose liking scores were only very weakly related to FN possessed characteristics not expected to induce arousal: high familiarity, sweetness, mild flavours, strong connections to national food cultures, or some combination of these factors. To overcome the limitations of the exploratory and data-driven approach, a range of suggestions for future research were proposed, notably direct measures of arousal, a more systematic selection of F&B items to be able to attribute the strength of the relationship between FN and liking to specific F&B characteristics. It would also be very pertinent to address the important issue of cross-cultural differences more systematically using, for example, items that are consumed (or avoided) in many different countries.

## Figures and Tables

**Table 1:** Overview of studies included in the research.

Study	Country	Date	FN Score M (SD)	F&B Stimuli (% Foods)	Number of Consumers	Age Range (Years)	% Male
1	USA	April 2019	30.5 (12.1)	26 (100)	1563	18–65	50
2	USA	June 2020	33.8 (12.4)	18 (100)	594	18–65	50
3	USA	June 2020	34.5 (11.5)	30 (67)	1522	18–65	49
4	Australia	June 2018	31.5 (10.8)	42 (81)	758	18–69	48
5	Australia	June 2020	33.3 (11.5)	18 (100)	1135	25–65	49
6	UK	June 2020	31.4 (11.7)	20 (95)	1514	18–65	47
7	Germany	June 2020	30.1 (10.0)	20 (100)	1040	18–65	49
8	Denmark	Nov. 2019	32.7 (10.7)	45 (84)	780	18–69	51

Notes: UK = United Kingdom; FN = food neophobia; M = mean and SD = standard deviation of summed FN score measured on the scale in Pliner and Hobden [5].

**Table 2:** Listing of F&B items (n = 197), sorted by group and regression coefficient (Coeff.) for effect of FN on liking/disliking. Names assigned to groups differentiate these on the level of negative effect of FN on F&B liking. For visual clarity, grey shading is used to differentiate groups. The final column shows average liking (1 = ‘dislike extremely’, 9 = ‘like extremely’).

Study	Country	F&B Name	Coeff.	Group Name	Liking
6	UK	Thai green chicken curry	−0.132	Very High	4.8
4	AU	Sushi	−0.128	Very High	6.3
4	AU	Thai green curry	−0.123	Very High	6.9
6	UK	Chilli chicken stir-fry	−0.122	Very High	5.2
8	DK	Sushi	−0.121	Very High	4.7
5	AU	Thai green curry	−0.120	Very High	5.4
1	USA	Sushi	−0.117	Very High	5.7
7	DE	Chilli chicken stir-fry	−0.116	Very High	5.7
5	AU	Sushi	−0.113	Very High	5.0
6	UK	Mild Indian curry (vegetarian)	−0.112	Very High	5.1
6	UK	Chilli con carne	−0.112	Very High	5.4
6	UK	Chicken korma	−0.109	Very High	5.4
4	AU	Prawn risotto	−0.109	Very High	6.0
8	DK	Thai meal	−0.106	Very High	6.1
6	UK	Spicy lamb meatballs	−0.106	Very High	4.9
5	AU	Prawn risotto	−0.104	Very High	5.2
3	USA	Spicy enchiladas	−0.103	Very High	5.5
6	UK	Lamb kebabs	−0.099	Very High	5.4
1	USA	Steamed mussels	−0.097	High	5.3
7	DE	Pasta with sundried tomato and garlic meat sauce	−0.095	High	5.8
3	USA	Vegetable chilli stir-fry	−0.095	High	5.0
6	UK	Chickpea salad (spicy)	−0.095	High	4.2
7	DE	Wholemeal pasta salad with chicken	−0.094	High	5.0
7	DE	Chicken and rice salad with spicy mayonnaise	−0.093	High	5.0
3	USA	Seafood pizza	−0.092	High	4.0
5	AU	Blue-vein cheese	−0.091	High	4.1
7	DE	Spinach and artichoke pizza	−0.091	High	4.4
1	USA	Fried oysters	−0.091	High	5.3
3	USA	Smoothie with avocado and almond milk	−0.090	High	4.3
5	AU	Spicy/hot chilli con carne	−0.090	High	4.9
4	AU	Bagel w/ avocado and cream cheese	−0.090	High	6.3
2	USA	Shrimp taco	−0.087	High	6.0
7	DE	Salsa black bean burger	−0.087	High	3.5
3	USA	Chai latte	−0.087	High	4.4
3	USA	Kale, cucumber and apple juice	−0.087	High	4.3
3	USA	Stuffed crust pizza with cheese, tomato and shrimp	−0.086	High	4.8
7	DE	Vegetarian pizza	−0.086	High	4.6
5	AU	Tuna steak	−0.086	High	5.0
1	USA	Salsa poached eggs	−0.086	High	5.0
3	USA	Lasagna made with meat substitutes from pea protein	−0.085	High	4.1
5	AU	Strong mustard	−0.084	High	4.7
3	USA	Lentil and broccoli “meat balls”	−0.082	High	4.1
3	USA	Vegan “meat balls” made with soy protein	−0.082	High	4.0
8	DK	Kebab with green salad	−0.082	High	6.6
5	AU	Rabbit ragu	−0.082	High	3.7
8	DK	Toasted rye bread with fried egg and avocado	−0.081	High	6.3
3	USA	Granola bar with coconut and chia seeds	−0.080	High	5.1
2	USA	Fried mushrooms	−0.080	High	6.0
3	USA	Oat milk with cocoa flavour	−0.080	High	4.4
2	USA	Root vegetable stew	−0.080	High	5.1
4	AU	Spinach and tomato omelette	−0.079	High	6.7
1	USA	Refried beans	−0.078	High	6.3
8	DK	Vegetarian pizza	−0.078	High	5.1
1	USA	Three cheese and chorizo omelette	−0.078	High	6.4
3	USA	Kombucha with ginger	−0.077	High	3.7
3	USA	Onion and beet salad	−0.077	High	3.9
3	USA	Baked salmon	−0.077	High	5.8
1	USA	Hot pastrami sandwich	−0.077	High	6.6
6	UK	Chicken fried rice	−0.076	High	6.3
3	USA	Apple, orange and kale juice	−0.076	High	5.1
5	AU	Smoked cheese	−0.075	High	3.7
1	USA	Eggs Benedict	−0.075	High	6.3
6	UK	Tuna pasta	−0.074	High	5.2
1	USA	Kidney bean salad	−0.074	High	5.0
3	USA	Wholemeal pasta with garlic and tomato sauce	−0.074	High	5.9
3	USA	Breakfast burrito	−0.073	High	6.2
2	USA	Seafood chowder	−0.073	High	5.7
8	DK	Roasted nuts	−0.072	High	6.6
8	DK	Pasta salad with feta cheese	−0.072	High	6.1
4	AU	Vegetable and bean hot pot	−0.072	High	6.5
6	UK	Soy milk	−0.071	High	3.5
3	USA	Vegetable pot pie	−0.071	High	5.4
6	UK	Sardines on toast	−0.070	Medium	3.8
8	DK	Mixed nuts with dried fruits	−0.070	Medium	6.0
2	USA	Corn chowder	−0.069	Medium	5.8
8	DK	Spinach and tomato omelette	−0.068	Medium	6.8
6	UK	Crackers with salmon pate	−0.068	Medium	4.4
7	DE	Stuffed bread with cheese and herbs	−0.068	Medium	6.1
8	DK	Vegetable juice	−0.067	Medium	4.9
3	USA	Zucchini brownie	−0.067	Medium	4.5
1	USA	Vegetable and bean casserole	−0.066	Medium	5.9
6	UK	Beef and beetroot sausages	−0.066	Medium	4.1
4	AU	Apple and kale juice	−0.066	Medium	5.3
1	USA	Baked salmon	−0.065	Medium	7.1
6	UK	Mixed green salad	−0.065	Medium	5.9
7	DE	Vegetarian meat loaf	−0.065	Medium	3.4
6	UK	Tuna steak	−0.065	Medium	5.5
2	USA	Lamb stew	−0.065	Medium	5.1
4	AU	Ham and tomato muffin	−0.064	Medium	6.3
5	AU	Pickled herring	−0.064	Medium	3.1
3	USA	Hot coffee	−0.063	Medium	6.3
2	USA	Lentil and beet soup	−0.063	Medium	4.6
7	DE	Ham and potato soup	−0.063	Medium	6.0
8	DK	Herbal tea	−0.063	Medium	4.5
4	AU	Caesar salad	−0.063	Medium	6.9
4	AU	Vegetarian sausages	−0.062	Medium	5.1
1	USA	Pickled beet and onion salad	−0.061	Medium	4.1
8	DK	Cheese fondue	−0.061	Medium	4.8
2	USA	Cream of mushroom soup	−0.061	Medium	5.7
1	USA	Breakfast burrito	−0.060	Medium	6.9
8	DK	Salmon and green salad	−0.058	Medium	6.6
4	AU	Frozen yoghurt	−0.058	Medium	6.2
7	DE	Chicken casserole	−0.058	Medium	6.2
3	USA	Club soda	−0.057	Medium	4.7
4	AU	Raw snack vegetables	−0.057	Medium	6.2
7	DE	Potato and lentil soup	−0.056	Medium	6.0
7	DE	Spicy red cabbage	−0.056	Medium	5.2
4	AU	Yoghurt	−0.055	Medium	7.1
4	AU	Egg mayonnaise sandwich	−0.055	Medium	6.4
2	USA	Burger with patty from 100% plant-based meat substitute	−0.055	Medium	5.0
4	AU	Mixed green salad	−0.055	Medium	7.0
6	UK	Dairy-free yoghurt	−0.054	Medium	4.4
8	DK	Beer	−0.054	Medium	5.3
8	DK	Sparkling water	−0.054	Medium	5.6
1	USA	Baked rabbit	−0.054	Medium	4.0
1	USA	Liver pate	−0.053	Medium	3.5
5	AU	Brussel sprouts	−0.053	Medium	5.0
2	USA	Veal burger	−0.053	Medium	4.9
4	AU	Hot coffee	−0.052	Medium	7.1
1	USA	Tripe and onions	−0.052	Medium	3.5
3	USA	Tossed green salad with red onions	−0.052	Medium	6.3
8	DK	Mixed grilled vegetables	−0.052	Medium	7.4
2	USA	Impossible™ burger (from plants)	−0.052	Medium	5.0
3	USA	Fish fingers	−0.052	Medium	5.3
8	DK	Stewed apples	−0.052	Medium	5.8
4	AU	Mixed raw nuts	−0.051	Medium	6.8
7	DE	Vegan bratwurst	−0.051	Medium	3.3
4	AU	Camomile tea	−0.051	Medium	5.2
4	AU	Iced coffee	−0.050	Medium	6.0
3	USA	Granola bar with insect flour	−0.050	Medium	3.1
6	UK	Savoury mince	−0.050	Medium	6.1
8	DK	Skyr with muesli	−0.050	Medium	5.1
4	AU	Croissant	−0.049	Medium	7.1
8	DK	Bun (Focaccia bread) with turkey, salad and dressing	−0.049	Medium	6.9
1	USA	Mixed green salad	−0.049	Medium	7.3
8	DK	Ham and cheese quiche	−0.048	Medium	6.9
2	USA	Lamb chops	−0.047	Low	5.6
7	DE	Bread and cheese	−0.047	Low	7.1
1	USA	Stewed prunes	−0.045	Low	3.4
7	DE	Herring fillet in tomato sauce	−0.044	Low	5.2
3	USA	Strawberry flavoured milk (from cows)	−0.043	Low	5.4
8	DK	Quiche with leek and bacon	−0.043	Low	7.1
8	DK	Muesli with milk	−0.042	Low	5.4
2	USA	Burger with patty from ground beef and vegetable blend (50:50)	−0.042	Low	5.4
4	AU	Cheese and vegemite sandwich	−0.042	Low	6.0
4	AU	Scrambled eggs	−0.041	Low	7.4
2	USA	Fried liver	−0.041	Low	3.9
5	AU	Dark chocolate	−0.040	Low	6.5
8	DK	White bread roll with ham and cheese	−0.040	Low	6.8
1	USA	Tuna salad sandwich	−0.039	Low	6.7
8	DK	Wholemeal bread with jam	−0.038	Low	6.4
8	DK	Fish cake on bread	−0.038	Low	7.0
3	USA	Strawberry and banana smoothie	−0.037	Low	6.8
4	AU	Sparkling water	−0.037	Low	5.7
7	DE	Spaghetti Bolognaise	−0.037	Low	7.4
8	DK	Rye bread with sliced meat	−0.036	Low	6.3
1	USA	Pickled pigs’ feet	−0.036	Low	2.7
6	UK	Pork and potato sausages	−0.036	Low	5.4
4	AU	Peanut butter sandwich	−0.035	Low	6.4
1	USA	Chilli cheese dog	−0.035	Low	6.6
6	UK	Chicken casserole	−0.034	Low	6.7
4	AU	Porridge/hot oatmeal	−0.033	Low	6.2
8	DK	Rye bread with cheese	−0.033	Low	7.0
4	AU	Ham and cheese muffin	−0.033	Low	6.6
2	USA	All-American beef stew	−0.032	Low	7.0
8	DK	Beef rissole and potato salad	−0.031	Low	7.5
4	AU	Instant noodles	−0.031	Low	6.0
4	AU	Fresh fruit salad	−0.031	Low	7.4
8	DK	Water	−0.030	Low	7.8
8	DK	Raw vegetables: tomato, cucumber, cauliflower, capsicum	−0.030	Low	7.5
4	AU	Lemon mousse tart	−0.028	Very Low	6.4
4	AU	Spaghetti Bolognaise	−0.027	Very Low	7.5
3	USA	Beef lasagna	−0.027	Very Low	7.3
1	USA	Cereal/muesli	−0.026	Very Low	6.4
4	AU	Banana	−0.026	Very Low	7.1
8	DK	Coffee	−0.026	Very Low	6.7
5	AU	Mild cheese	−0.026	Very Low	6.7
8	DK	Warm liver pate with mushrooms	−0.025	Very Low	7.4
8	DK	Porridge/hot oatmeal	−0.024	Very Low	5.6
8	DK	Fruit salad	−0.024	Very Low	7.4
7	DE	Meat loaf	−0.023	Very Low	6.8
4	AU	Cereal/muesli	−0.023	Very Low	6.9
4	AU	Cold sliced meats	−0.022	Very Low	6.8
1	USA	Blueberry muffins	−0.022	Very Low	7.5
8	DK	Broth with vegetables and meat balls	−0.022	Very Low	7.2
5	AU	White rice	−0.020	Very Low	6.7
8	DK	Soft boiled egg with bread	−0.020	Very Low	7.5
5	AU	Garlic bread	−0.020	Very Low	7.5
4	AU	Ham and cheese sandwich	−0.018	Very Low	7.1
1	USA	Instant noodles	−0.018	Very Low	6.2
2	USA	Chicken sandwich	−0.016	Very Low	7.7
3	USA	Spaghetti with tomato sauce	−0.015	Very Low	7.6
4	AU	Fruit juice	−0.014	Very Low	7.1
1	USA	Lasagna	−0.014	Very Low	8.0
8	DK	Spaghetti Bolognaise	−0.013	Very Low	8.0
5	AU	Apple	−0.011	Very Low	7.2
8	DK	Danish pastry	−0.002	Very Low	6.7

Notes: The group of F&B items with positive regression coefficients (n = 2) is not shown. The F&B items with non-significant models (n = 20) are not shown. AU = Australia, DE = Germany, DK = Denmark.

**Table 3:** Derived categories of F&B characteristics and their presence/absence in the five groups categorising the varying negative effect of FN of F&B liking (“very high”, “high”, “medium”, “low” and “very low”). The categories of F&B characteristics should be seen in the context of those above it and refer to F&B characteristics not previously captured. The exemplar F&B items illustrate the associated category of F&B item characteristics, but items may also fit into other categories without this being shown.

Categories of F&B Characteristics	Degree of Negative Effect of FN on Liking					Exemplars
	Very High	High	Medium	Low	Very Low	
From other cultures	x	x				Thai green curry, chicken korma
Shellfish/Sushi	x	x				Prawn risotto, sushi
Chilli/Spicy	x	x	x			Chilli con carne, spicy lamb meatballs
Strong flavour		x	x			Blue cheese, kombucha with ginger, beet and onion salad
Unusual meat/Offal		x	x			Rabbit, veal, liver, tripe and onions
Fish		x	x	x		Sardines on toast, tuna steak, baked salmon
Reduced familiarity		x	x	x		Three cheese and chorizo omelette, cheese fondue
Familiar but often disliked		x	x	x		Brussel sprouts, dark chocolate, coffee, porridge, vegetable juice
Familiar w/ novel ingredients		x	x			Insect flour, chia seeds, oat milk, 100% plant-based meat, soy protein
Familiar w/ unusual ingredient/aspect		x	x			Apple and kale juice, zucchini brownie, stewed apples
Beans/Legumes		x	x			Vegetable and bean casserole, kidney bean salad
Vegetables/Salad			x			Mixed green salad, mixed grilled vegetables, raw snack vegetables
Soup			x			Corn chowder, ham and potato, cream of mushroom
Non-alcoholic beverages			x	x	x	Water, fruit juice, club soda, strawberry and banana smoothie
Familiar hot meals w/ meat			x	x	x	Spaghetti Bolognaise, lasagna, meat loaf
Familiar and grain-based				x	x	Ham and cheese sandwich, rye bread with cheese
Familiar desserts/cakes					x	Blueberry muffins, Danish pastry
Fruit					x	Apple, banana
Mild flavour					x	Mild cheese, white rice

## Data Availability

The data presented in this study are available on request from the corresponding author.

## Supplementary Materials

The following are available online at www.mdpi.com/article/10.3390/nu13103657/s1. Table S1a–h: Summary of participant characteristics by study; Table S2: F&B names used in Germany (Study 7) and Denmark (Study 8) in original languages; Table S3: Regression statistics for F&B names included in the research and average hedonic scores (n = 219); Figure S1: Distribution of FN scores in Study 1; Table S4: Overview of the six groups of F&B names with variable impact of FN on liking; Figure S2: Plot of average F&B liking as a function of FN for selected F&B names.

## References

[1] Cashdan E. (1998). Adaptiveness of food learning and food aversions in children. Soc. Sci. Inf..

[2] Hursti U.-K.K., Sjoden P.-O. (1997). Food and General Neophobia and their Relationship with Self-Reported Food Choice: Familial Resemblance in Swedish Families with Children of Ages 7–17 Years. Appetite.

[3] Russell C.G., Worsley A. (2008). A Population-based Study of Preschoolers’ Food Neophobia and Its Associations with Food Preferences. J. Nutr. Educ. Behav..

[4] Skinner J.D., Carruth B.R., Wendy B., Ziegler P.J. (2002). Children’s food preferences: A longitudinal analysis. J. Am. Diet. Assoc..

[5] Pliner P., Hobden K. (1992). Development of a scale to measure the trait of food neophobia in humans. Appetite.

[6] Jaeger S.R., Rasmussen M.A., Prescott J. (2017). Relationships between food neophobia and food intake and preferences: Findings from a sample of New Zealand adults. Appetite.

[7] Monteleone E., Spinelli S., Dinnella C., Endrizzi I., Laureati M., Pagliarini E., Sinesio F., Gasperi F., Torri L., Aprea E. (2017). Exploring influences on food choice in a large population sample: The Italian Taste project. Food Qual. Prefer..

[8] Meiselman H.L., King S.C., Gillette M. (2010). The demographics of neophobia in a large commercial US sample. Food Qual. Prefer..

[9] Knaapila A., Silventoinen K., Broms U., Rose R.J., Perola M., Kaprio J., Tuorila H.M. (2011). Food Neophobia in Young Adults: Genetic Architecture and Relation to Personality, Pleasantness and Use Frequency of Foods, and Body Mass Index—A Twin Study. Behav. Genet..

[10] Asperin A.E., Phillips W.J., Wolfe K. Exploring Food Neophobia and Perceptions of Ethnic Foods: The Case of Chinese and Thai Cuisines. Proceedings of the International CHRIE Conference-Refereed Track 2011 ICHRIE Conference.

[11] Henriques A.S., King S.C., Meiselman H.L. (2009). Consumer segmentation based on food neophobia and its application to product development. Food Qual. Prefer..

[12] De Toffoli A., Spinelli S., Monteleone E., Arena E., Di Monaco R., Endrizzi I., Gallina Toschi T., Laureati M., Napolitano F., Torri L. (2019). Influences of Psychological Traits and PROP Taster Status on Familiarity with and Choice of Phenol-Rich Foods and Beverages. Nutrients.

[13] Laureati M., Spinelli S., Monteleone E., Dinnella C., Prescott J., Cattaneo C., Proserpio C., De Toffoli A., Gasperi F., Endrizzi I. (2018). Associations between food neophobia and responsiveness to “warning” chemosensory sensations in food products in a large population sample. Food Qual. Prefer..

[14] Mustonen S., Oerlemans P., Tuorila H. (2012). Familiarity with and affective responses to foods in 8-11-year-old children. The role of food neophobia and parental education. Appetite.

[15] Raudenbush B., Frank R.A. (1999). Assessing Food Neophobia: The Role of Stimulus Familiarity. Appetite.

[16] Siegrist M., Hartmann C., Keller C. (2013). Antecedents of food neophobia and its association with eating behavior and food choices. Food Qual. Prefer..

[17] Tuorila H., Lahteenmaki L., Pohjalainen L., Lotti L. (2001). Food neophobia among the Finns and related responses to familiar and unfamiliar foods. Food Qual. Prefer..

[18] Pliner P., Pelchat M.L. (1991). Neophobia in humans and the special status of foods of animal origin. Appetite.

[19] Rozin E., Rozin P. (1981). Culinary themes and variations. Nat. Hist..

[20] Knaapila A., Tuorila H., Silventoinen K., Keskitalo K., Kallela M., Wessman M., Peltonen L., Cherkas L.F., Spector T.D., Perola M. (2007). Food neophobia shows heritable variation in humans. Physiol. Behav..

[21] Duffy E. (1957). The psychological significance of the concept of “arousal” or “activation”. Psychol. Rev..

[22] Russell J.A., Weiss A., Mendelsohn G.A. (1989). Affect Grid: A Single-Item Scale of Pleasure and Arousal. J. Personal. Soc. Psychol..

[23] Berlyne D.E. (1970). Novelty, complexity, and hedonic value. Percept. Psychophys..

[24] Pliner P., Pelchat M., Grabski M. (1993). Reduction of neophobia in humans by exposure to novel foods. Appetite.

[25] Raudenbush B., Capiola A. (2012). Physiological responses of food neophobics and food neophilics to food and non-food stimuli. Appetite.

[26] Coulthard H., Blissett J. (2009). Fruit and vegetable consumption in children and their mothers. Moderating effects of child sensory sensitivity. Appetite.

[27] Raudenbush B., Schroth F., Reilley S., Frank R.A. (1998). Food neophobia, odor evaluation and exploratory sniffing behavior. Appetite.

[28] Giacalone D., Duerlund M., Boegh-Petersen J., Bredie W.L., Frost M.B. (2014). Stimulus collative properties and consumers’ flavor preferences. Appetite.

[29] Spinelli S., De Toffoli A., Dinnella C., Monteleone E., Gavazzi G., Prescott J., Pierguidi L. Individual differences in arousal induced by taste quality, intensity and valence. Proceedings of the 9th European Conference on Sensory and Consumer Science.

[30] Olabi A., Neuhaus T., Bustos R., Cook-Camacho M., Corvi T., Abdouni L. (2015). An investigation of flavor complexity and food neophobia. Food Qual. Prefer..

[31] Pliner P., Melo N. (1997). Food Neophobia in Humans: Effects of Manipulated Arousal and Individual Differences in Sensation Seeking. Physiol. Behav..

[32] Zuckerman M., Kolin E.A., Price L., Zoob I. (1964). Development of a Sensation-Seeking Scale. J. Consult. Psychol..

[33] Tuorila H., Meiselman H.L., Bell R., Cardello A.V., Johnson W. (1994). Role of sensory and cognitive information in the enhancement of certainty and liking for novel and familiar foods. Appetite.

[34] Moding K.J., Stifter C.A. (2016). Temperamental approach/withdrawal and food neophobia in early childhood: Concurrent and longitudinal associations. Appetite.

[35] Alley T.R., Potter K.A., Preedy V., Watson R., Martin C. (2011). Food Neophobia and Sensation Seeking. Handbook of Behavior, Food and Nutrition.

[36] Brown S.D., Harris G. (2012). A Theoretical Proposal for a Perceptually Driven, Food-Based Disgust that Can Influence Food Acceptance During Early Childhood. Int. J. Child Health Nutr..

[37] Spinelli S., De Toffoli A., Dinnella C., Laureati M., Pagliarini E., Bendini A., Braghieri A., Gallina Toschi T., Sinesio F., Torri L. (2018). Personality traits and gender influence liking and choice of food pungency. Food Qual. Prefer..

[38] Mascarello G., Pinto A., Rizzoli V., Tiozzo B., Crovato S., Ravarotto L. (2020). Ethnic Food Consumption in Italy: The Role of Food Neophobia and Openness to Different Cultures. Foods.

[39] Farrow C.V., Coulthard H. (2012). Relationships between sensory sensitivity, anxiety and selective eating in children. Appetite.

[40] Zickgraf H.F., Elkins A. (2018). Sensory sensitivity mediates the relationship between anxiety and picky eating in children/ adolescents ages 8–17, and in college undergraduates: A replication and age-upward extension. Appetite.

[41] Maratos F.A., Staples P. (2015). Attentional biases towards familiar and unfamiliar foods in children. The role of food neophobia. Appetite.

[42] Cisler J.M., Koster E.H. (2010). Mechanisms of attentional biases towards threat in anxiety disorders: An integrative review. Clin. Psychol. Rev..

[43] Jaeger S.R., Chheang S.L., Jin D., Ryan G., Worch T. (2021). The negative influence of food neophobia on food and beverage liking: Time to look beyond extreme groups analysis?. Food Qual. Prefer..

[44] Ritchey P.N., Frank R.A., Hursti U.-K., Tuorila H. (2003). Validation and cross-national comparison of the food neophobia scale (FNS) using confirmatory factor analysis. Appetite.

[45] Peryam D.R., Pilgrim F.J. (1957). Hedonic scale method of measuring food preferences. Food Technol..

[46] Jaeger S.R., Roigard C.M., Hunter D.C., Worch T. (2020). Importance of food choice motives vary with degree of food neophobia. Appetite.

[47] Jaeger S.R., Roigard C.M., Le Blond M., Hedderley D.I., Giacalone D. (2019). Perceived situational appropriateness for foods and beverages: Consumer segmentation and relationship with stated liking. Food Qual. Prefer..

[48] Jaeger S.R., Prescott J., Worch T. (2021). Food neophobia modulates importance of food choice motives: Replication, extension and behavioural validation. Food Qual. Prefer..

[49] Tavakol M., Dennick R. (2011). Making sense of Cronbach’s alpha. Int. J. Med. Educ..

[50] Schickenberg B., van Assema P., Brug J., de Vries N.K. (2007). Are the Dutch acquainted with and willing to try healthful food products? The role of food neophobia. Public Health Nutr..

[51] Verbeke W. (2005). Consumer acceptance of functional foods: Socio-demographic, cognitive and attitudinal determinants. Food Qual. Prefer..

[52] Jacobs S., Sioen I., Pieniak Z., De Henauw S., Maulvault A.L., Reuver M., Fait G., Cano-Sancho G., Verbeke W. (2015). Consumers’ health risk-benefit perception of seafood and attitude toward the marine environment: Insights from five European countries. Environ. Res..

[53] Burger J., Gochfeld M. (2009). Perceptions of the risks and benefits of fish consumption: Individual choices to reduce risk and increase health benefits. Environ. Res..

[54] Pieniak Z., Verbeke W., Scholderer J., Brunsø K., Ottar Olsen S. (2008). Impact of consumers’ health beliefs, health involvement and risk perception on fish consumption. Br. Food J..

[55] Rozin P., Zentall T.R., Galef B.G. (1988). Social learning about food by Humans. Social Learning: Psychological and Biological Perspectives.

[56] Sulmont-Rossé C., Chabanet C., Issanchou S., Köster E.P. (2008). Impact of the arousal potential of uncommon drinks on the repeated exposure effect. Food Qual. Prefer..

[57] Kraig B., Sen C.T. (2013). Street Food around the World: An Encyclopedia of Food and Culture.

[58] Ireland J., van Erp-Baart A., Charrondière U., Møller A., Smithers G., Trichopoulou A. (2002). Selection of a food classification system and a food composition database for future food consumption surveys. Eur. J. Clin. Nutr..

[59] Feldman Barrett L., Russell J.A. (1998). Independence and bipolarity in the structure of current affect. J. Personal. Soc. Psychol..

[60] den Uijl L.C., Jager G., Zandstra E.H., de Graaf C., Kremer S. (2016). Self-reported food-evoked emotions of younger adults, older normosmic adults, and older hyposmic adults as measured using the PrEmo2 tool and the Affect Grid. Food Qual. Prefer..

