# Supplementary material for: Variations in the Strength of Association between Food Neophobia and Food and Beverage Acceptability: A Data-Driven Exploratory Study of an Arousal Hypothesis
Source: Nutrients. 2021 Oct 19;13(10):3657. doi: 10.3390/nu13103657 (PMC8540144; doi:10.3390/nu13103657)

Supplementary Materials

Table S1a. Summary participant characteristics for Study 1 (USA: n=1563), shown as percentages.

	Total sample
**Gender**	
Men	50
Women	50
**Age in years**	
18-39	50
40-65	50
**Education level***	
Less than high school	1
High school or GED	15
Associate/technical	10
Some college	22
College	36
Postgraduate	16
**Household member***	
No-one, I live alone	22
Spouse/partner	55
Child/ren aged under 18	33
Child/ren aged over 18	9
Parents	12
Housemate/s	6
Other	3
**Household income (USD/year)**	
Less than $30,000	21
$30,000 - $54,999	24
$55,000 - $84,999	21
$85,000 -$99,999	10
$100,000 or more	21
Prefer not to answer	3
**Ethnicity***	
White/Caucasian	78
Black/African American	9
Spanish/Hispanic/Latino	8
Asian	7
Pacific Islander	0
Native American	2
Other	1

Note. *) Total responses are >100% as consumers may select multiple options. GED = GED is a high school equivalency diploma. 'Some college' refers to initiated but incomplete degree.

Table S1b. Summary participant characteristics for Study 2 (USA: n=594), shown as percentages.

	Total sample
**Gender**	
Men	50
Women	50
**Age in years**	
18-39	50
40-65	50
**Education level***	
Less than high school	1
High school or GED	20
Associate/technical	10
Some college	16
College	33
Postgraduate	19
**Household member***	
No-one, I live alone	21
Spouse/partner	56
Child/ren aged under 18	34
Child/ren aged over 18	11
Parents	13
Housemate/s	3
Other	4
**Household size**	
1 person	21
2 people	32
3 people	20
4 people	19
5 or more people	8
**Employment**	
Working full-time	49
Working part-time	13
No-paid work/home duties	7
Student	4
Unemployed	13
Retired	13
Other	3
**Household income (USD/year)**	
Less than $30,000	20
$30,000 - $59,999	21
$60,000 - $79,999	14
$80,000 - $99,999	16
$100,000 - $149,000	15

$150,000 or more	11
Prefer not to answer	2
**Ethnicity***	
White/Caucasian	81
Black/African American	10
Spanish/Hispanic/Latino	6
Asian	5
Pacific Islander	1
Native American	2
Middle Eastern/North African	0
Other	1
Prefer not to answer	0

Note. *) Total responses are >100% as consumers may select multiple options. GED = GED is a high school equivalency diploma. 'Some college' refers to initiated but incomplete degree.

Table S1c. Summary participant characteristics for Study 3 (USA n=1522), shown as percentages.

	Total sample
**Gender**	
Men	49
Women	51
**Age in years**	
18-39	49
40-65	51
**Education level***	
Less than high school	1
High school or GED	17
Associate/technical	9
Some college	20
College	35
Postgraduate	18
**Household member***	
No-one, I live alone	20
Spouse/partner	54
Child/ren aged under 18	30
Child/ren aged over 18	10
Parents	17
Housemate/s	4
Other	4
**Household size**	
1 person	20
2 people	35
3 people	21
4 people	15
5 or more people	9
**Employment**	
Working full-time	52
Working part-time	12
No-paid work/home duties	5
Student	6
Unemployed	10
Retired	10
Other	2
Disabled	4
**Household income (USD/year)**	
Less than $30,000	19
$30,000 - $59,999	25
$60,000 - $79,999	14
$80,000 - $99,999	15

$100,000 - $149,000	14
$150,000 or more	10
Prefer not to answer	3
**Ethnicity***	
White/Caucasian	78
Black/African American	8
Spanish/Hispanic/Latino	9
Asian	7
Pacific Islander	1
Native American	1
Middle Eastern/North African	1
Other	1
Prefer not to answer	1

Note. *) Total responses are >100% as consumers may select multiple options. GED = GED is a high school equivalency diploma. 'Some college' refers to initiated but incomplete degree.

Table S1d. Summary participant characteristics for Study 4 (Australia n=758), shown as percentages.

	Total sample
**Gender**	
Men	49
Women	51
**Age in years**	
18-29	23
30-39	21
40-49	20
50-59	20
60-69	16
**Education level**	
Year 12 or less	23
Diploma or vocation certificate	27
University degree	50
**Household member***	
No-one, I live alone	18
Spouse/partner	62
Child/ren aged under 18	30
Child/ren aged over 18	17
Parents	9
Housemate/s	5
Other	2
**Household size**	
1 person	19
2 people	33
3 people	20
4 people	19
5 or more people	8
**Household income (AUD/year)**	
Less than $30,000	10
$30,000 – $54,999	15
$55,000 – $84,999	17
$85,000 or more	48
Prefer not to answer	10

Note. *) Total responses are >100% as consumers may select multiple options.

Table S1e. Summary participant characteristics for Study 5 (Australia n=1135), shown as percentages.

	Total sample
**Gender**	
Men	49
Women	51
**Age in years**	
25-29	8
30-35	15
36-40	12
41-45	13
46-50	10
51-55	10
56-60	16
61-65	17
**Education level**	
Year 12 or less	26
Diploma or vocation certificate	28
University degree	45
Other	1
**Household size**	
1 person	18
2 people	35
3 people	22
4 people	18
5 or more people	7
**Employment**	
Working full-time	49
Working part-time	18
No-paid work/home duties	13
Student	1
Unemployed	11
Other	8
**Household income (AUD/year)**	
Less than $60,000	26
$60,000-$79,999	16
$80,000-$99,999	14
$100,000-$149,999	21
$150,000-$199,999	9
$200,000 +	7
Prefer not to answer	7

Table S1f. Summary participant characteristics for Study 6 (UK n=1514), shown as percentages.

	Total sample
**Gender**	
Men	47
Women	53
**Age in years**	
18-39	48
40-65	52
**Education level**	
Lower than high school	1
GCSE's	19
A-levels	16
Associate/technical	6
College	32
Postgraduate	21
Other	3
**Household member***	
No-one, I live alone	19
Spouse/partner	60
Child/ren aged under 18	31
Child/ren aged over 18	11
Parents	13
Housemate/s	3
Other	2
**Household size**	
1 person	19
2 people	35
3 people	21
4 people	18
5 or more people	6
**Employment**	
Working full-time	55
Working part-time	16
No-paid work/home duties	7
Student	4
Unemployed	8
Retired	7
Other	1
Disabled	2
**Household income (GBP/year)**	
Less than £10,000	6
£10,000-£19,999	15
£20,000-£29,999	18
£30,000-£39,999	16
£40,000-£49,999	19

More than £50,000	22
Prefer not to answer	4
**Ethnicity***	
British	88
Chinese	2
Indian	2
Pakistani	1
Black African	1
Black Caribbean	1
Bangladeshi	1
Other	4
Prefer not to answer	1

Note. *) Total responses are >100% as consumers may select multiple options.

Table S1g. Summary participant characteristics for Study 7 (Germany n=1040), shown as percentages.

	Total sample
**Gender**	
Men	49
Women	51
**Age in years**	
18-39	50
40-65	50
**Education level**	
No secondary school certificate	0
Secondary school certificate (Hauptschule)	3
Secondary school certificate (Realschule)	10
Secondary school certificate (Gymnasium)	13
Completed vocational training	38
Polytechnic degree	11
University degree	25
No information	0
Other	0
**Household member***	
No-one, I live alone	29
Spouse/partner	55
Child/ren aged under 18	25
Child/ren aged over 18	7
Parents	8
Housemate/s	5
Other	0
**Household size**	
1 person	29
2 people	36
3 people	18
4 people	12
5 or more people	4
**Employment**	
Working full-time	57
Working part-time	13
No-paid work/home duties	5
Student	8
Unemployed	5
Retired	8
Other	1
Disabled	3
**Household income (Euro/year)**	

Less than €20,000	19
€20,000 - €29,999	15
€30,000-€39,999	16
€40,000-€49,999	16
More than €50,000	30
Prefer not to answer	4

Note. *) Total responses are >100% as consumers may select multiple options.

Table S1h. Summary participant characteristics for Study 8 (Denmark n=780), shown as percentages.

	Total sample
**Gender**	
Men	51
Women	49
**Age in years**	
18-29	21
30-39	18
40-49	21
50-59	21
60-69	20
**Education level**	
Primary school	12
High school	13
Skill school	15
Short higher education (≤2 years)	14
Medium higher education (3-5 years)	30
Long higher education (≥5 years)	15
Other	1
**Household size**	
1 person	33
2 people	35
3 people	16
4 people	12
5 or more people	5
**Employment**	
Working full-time	46
Working part-time	9
No-paid work/home duties	3
Student	11
Unemployed	12
Other	19
**Household income (DKK/year)**	
Less than DKK 200.000	24
DKK 200.001 – DKK 350.000	26
DKK 350.001 – DKK 550.000	23
DKK 550.001 – DKK 750.000	6
DKK 750.001 – DKK 950.000	4
DKK 950.001 or more	3
Prefer to not answer	15

Table S2. F&B names in German (Study 7) and Danish (Study 8) used during data collection (right-most column).

#	English name	German or Danish name
7	Bratwurst	Bratwurst
7	Bread and cheese	Brot und Käse
7	Chicken casserole	Hähnchen-Auflauf
7	Ham and potato soup	Kartoffelsuppe mit Schinkeneinlage
7	Meat loaf	Hackbraten
7	Pasta with sundried tomato and garlic meat sauce	Nudeln mit sonnengetrockneten Tomaten und Knoblauch-Fleischsauce
7	Potato and lentil soup	Kartoffel-Linsen-Suppe
7	Salsa black bean burger	Vegetarischer Burger mit Bratling aus Salsa und schwarzen Bohnen
7	Sauerkraut	Sauerkraut
7	Spaghetti Bolognaise	Spaghetti Bolognese
7	Spinach and artichoke pizza	Pizza mit Spinat und Artischocken
7	Stuffed bread with cheese and herbs	Gefülltes Brot mit Kaese und Kräutern?
7	Vegan bratwurst	Vegane Bratwurst
7	Vegetarian meat loaf	Vegetarischer Hackbrate
7	Vegetarian pizza	Vegetarische Pizza
7	Wholemeal pasta salad with chicken	Vollkorn Nudelsalat mit Hühnchen
8	Banana	Banan
8	Beef risole and potato salad	Hakkebøf med kartoffelsalat
8	Beer	Øl
8	Broth with vegetables and meat balls	Klar suppe med grøntsager og kødboller
8	Cheese fondue	Ostefondue
8	Coca Cola	Coca Cola
8	Coffee	Kaffe
8	Danish pastry	Wienerbrød
8	French fries	Pomfritter
8	French hotdog wo/ dressing	Pølsehorn
8	Fruit salad	Frugtsalat

8	Ham and cheese quiche	Skinketærte med ost
8	Herbal tea	Urtete
8	Kebab with green salad	Kebab med grøn salat
8	Milk and rice porridge	Risengrød
8	Mixed grilled vegetables	Ovnstegte grøntsager
8	Mixed nuts with dried fruits	Nødde blanding med tørrede frugter
8	Muesli with milk	Mysli med mælk
8	Orange juice	Appelsinjuice
8	Pasta salad with feta cheese	Pastasalat med fetaost
8	Porridge/Hot oatmeal	Havregrød
8	Quiche with leek and bacon	Porretærte med bacon
8	Raw vegetables: tomato, cucumber, cauliflower, capsicum	Rå grøntsager (tomat, agurk, blomkål, peberfrugt)
8	Roasted nuts	Ristede nødder
8	Ryebread with cheese	Rugbrød med ost
8	Ryebread with sliced meat	Rugbrød med rullepølse og sky
8	Bun (Focaccia bread) with turkey, salad and dressing	Focaccia bolle med kalkun, salat og dressing
8	Softboiled egg with bread	Blødkogt æg med brød
8	Spaghetti Bolognese	Spaghetti med kødsovs
8	Sparkling water	Dansk vand (med brus)
8	Spinach and tomato omelette	Omelet med spinat og tomat
8	Stewed apples	Æblegrød
8	Thai meal	Thai-mad
8	Toasted ryebread with fried egg and avocado	Ristet rugbrød med spejlæg og avocado
8	Vegetable juice	Grøntsagsjuice
8	Vegetarian pizza	Vegetar pizza
8	Warm liverpate with mushrooms	Lun leverpostej med champignon og bacon
8	Water	Vand
8	White bread roll with ham and cheese	Rundstykke med skinke og ost
8	Wholemeal bread with jam	Grovbrød med syltetøj
8	Skyr with muesli	Skyr med mysli

Table S3. List of F&B stimuli used in the research and key statistics following linear regression to establish the effect of FN on F&B liking/disliking. Shown are values for model goodness-of-fit (R^2) (where all models are significant at $p < 0.05$ unless indicated by 'ns' superscript) followed by regression coefficients (b) which are given with corresponding 95% confidence intervals (CI) as lower and upper bounds. The second to last column shows the intercept value for the linear regression (a), while the final column shows average degree of liking/disliking across all participants in the study (1='dislike extremely, 9='like extremely'). F&B names are shown by study and sorted by regression coefficient value. Grey shading used to differentiate between studies.

Study	F&B name*	R^2	b	b 95% CI lower	b 95% CI upper	a	Average liking
1	Sushi	0.93	-0.117	-0.128	-0.106	9.2	5.7
1	Steamed mussels	0.90	-0.097	-0.108	-0.085	8.2	5.3
1	Fried oysters	0.81	-0.091	-0.106	-0.075	8.0	5.3
1	Salsa poached eggs	0.86	-0.086	-0.098	-0.074	7.5	5.0
1	Refried beans	0.79	-0.078	-0.092	-0.065	8.6	6.3
1	Three cheese and chorizo omelette	0.80	-0.078	-0.091	-0.064	8.7	6.4
1	Hot pastrami sandwich	0.72	-0.077	-0.093	-0.060	8.8	6.6
1	Eggs Benedict	0.87	-0.075	-0.086	-0.065	8.5	6.3
1	Kidney bean salad	0.80	-0.074	-0.087	-0.062	7.1	5.0
1	Vegetable and bean casserole	0.76	-0.066	-0.079	-0.053	7.8	5.9
1	Baked salmon	0.72	-0.065	-0.080	-0.051	9.0	7.1
1	Pickled beet and onion salad	0.75	-0.061	-0.074	-0.049	5.8	4.1
1	Breakfast burrito	0.83	-0.060	-0.070	-0.050	8.7	6.9
1	Baked rabbit	0.74	-0.054	-0.065	-0.042	5.6	4.0
1	Liver pate	0.67	-0.053	-0.066	-0.040	5.0	3.5
1	Tripe and onions	0.74	-0.052	-0.063	-0.041	5.0	3.5
1	Mixed green salad	0.74	-0.049	-0.059	-0.039	8.8	7.3
1	Stewed prunes	0.66	-0.045	-0.056	-0.034	4.6	3.4
1	Tuna salad sandwich	0.42	-0.039	-0.056	-0.023	7.8	6.7
1	Pickled pigs feet	0.58	-0.036	-0.047	-0.025	3.7	2.7

1	Chilli cheese dog	0.40	-0.035	-0.050	-0.020	7.6	6.6
1	Cereal/muesli	0.48	-0.026	-0.036	-0.017	7.2	6.4
1	Blueberry muffins	0.28	-0.022	-0.034	-0.010	8.2	7.5
1	Instant noodles[2]	0.27	-0.018	-0.028	-0.008	6.7	6.2
1	Lasagna	0.27	-0.014	-0.022	-0.006	8.4	8.0
1	Chicken noodle soup	0.10 [ns]	-0.008	-0.017	0.000	7.6	7.4
2	Shrimp taco	0.68	-0.087	-0.108	-0.067	8.7	6.0
2	Fried mushrooms	0.62	-0.080	-0.101	-0.058	8.4	6.0
2	Root vegetable stew	0.71	-0.080	-0.097	-0.062	7.6	5.1
2	Seafood chowder	0.57	-0.073	-0.095	-0.051	8.0	5.7
2	Corn chowder	0.63	-0.069	-0.087	-0.051	7.9	5.8
2	Lamb stew	0.64	-0.065	-0.081	-0.048	7.1	5.1
2	Lentil and beet soup	0.57	-0.063	-0.082	-0.045	6.5	4.6
2	Cream of mushroom soup	0.52	-0.061	-0.081	-0.041	7.6	5.7
2	Burger with patty from 100% plant-based meat substitute	0.44	-0.055	-0.076	-0.034	6.6	5.0
2	Veal burger	0.52	-0.053	-0.070	-0.036	6.7	4.9
2	Impossible™ burger (from plants)	0.40	-0.052	-0.074	-0.030	6.4	5.0
2	Lamb chops	0.41	-0.047	-0.067	-0.028	7.1	5.6
2	Burger with patty from ground beef and vegetable blend (50:50)	0.31	-0.042	-0.063	-0.021	6.6	5.4
2	Fried liver	0.35	-0.041	-0.060	-0.022	4.9	3.9
2	All-American beef stew	0.22	-0.032	-0.052	-0.011	8.0	7.0
2	Chicken sandwich	0.12	-0.016	-0.031	-0.001	8.2	7.7
2	Fried eggs	0.07 [ns]	-0.014	-0.032	0.004	7.4	7.0
2	Burger with patty from 100% ground beef	0.05 [ns]	-0.007	-0.019	0.004	8.0	7.7
3	Spicy enchiladas	0.86	-0.103	-0.114	-0.091	9.5	5.5
3	Vegetable chilli stir-fry	0.78	-0.095	-0.109	-0.082	8.8	5.0
3	Seafood pizza	0.83	-0.092	-0.103	-0.081	7.6	4.0
3	Smoothie with avocado and almond milk[B]	0.78	-0.090	-0.103	-0.077	7.8	4.3
3	Chai latte[B]	0.78	-0.087	-0.099	-0.074	7.8	4.4
3	Kale, cucumber and apple juice[B]	0.79	-0.087	-0.099	-0.075	7.7	4.3

3	Stuffed crust pizza with cheese, tomato and shrimp	0.81	-0.086	-0.097	-0.075	8.2	4.8
3	Lasagna made with meat substitutes from pea protein	0.78	-0.085	-0.097	-0.073	7.4	4.1
3	Lentil and broccoli "meat balls"	0.75	-0.082	-0.095	-0.070	7.3	4.1
3	Vegan "meat balls" made with soy protein	0.79	-0.082	-0.093	-0.071	7.2	4.0
3	Granola bar with coconut and chia seeds	0.63	-0.080	-0.097	-0.064	8.2	5.1
3	Oat milk with cocoa flavour[B]	0.74	-0.080	-0.092	-0.067	7.5	4.4
3	Baked salmon[2]	0.64	-0.077	-0.092	-0.061	8.7	5.8
3	Kombucha with ginger*	0.77	-0.077	-0.088	-0.066	6.7	3.7
3	Onion and beet salad	0.74	-0.077	-0.089	-0.065	6.9	3.9
3	Apple, orange and kale juice[B]	0.75	-0.076	-0.087	-0.064	8.1	5.1
3	Wholemeal pasta with garlic and tomato sauce	0.65	-0.074	-0.088	-0.059	8.8	5.9
3	Breakfast burrito	0.74	-0.073	-0.085	-0.062	9.0	6.2
3	Vegetable pot pie	0.63	-0.071	-0.085	-0.056	8.2	5.4
3	Zucchini brownie	0.67	-0.067	-0.079	-0.054	7.1	4.5
3	Hot coffee[B]	0.55	-0.063	-0.079	-0.048	8.8	6.3
3	Club soda*	0.66	-0.057	-0.068	-0.046	6.9	4.7
3	Fish fingers	0.52	-0.052	-0.065	-0.038	7.3	5.3
3	Tossed green salad with red onions	0.50	-0.052	-0.066	-0.038	8.3	6.3
3	Granola bar with insect flour	0.53	-0.050	-0.063	-0.037	5.0	3.1
3	Strawberry flavoured milk (from cows)	0.41	-0.043	-0.056	-0.029	7.1	5.4
3	Strawberry and banana smoothie	0.37	-0.037	-0.050	-0.024	8.3	6.8
3	Beef lasagna	0.26	-0.027	-0.039	-0.015	8.4	7.3
3	Spaghetti with tomato sauce	0.24	-0.015	-0.022	-0.008	8.2	7.6
3	Chocolate cake	0.01 [ns]	-0.002	-0.012	0.007	7.9	7.8
4	Sushi	0.83	-0.128	-0.144	-0.112	10.2	6.3
4	Thai green curry	0.76	-0.123	-0.141	-0.104	10.5	6.9
4	Prawn risotto	0.73	-0.109	-0.126	-0.091	9.2	6.0
4	Bagel with avocado and cream cheese	0.58	-0.090	-0.111	-0.069	9.0	6.3
4	Spinach and tomato omelette	0.48	-0.079	-0.101	-0.057	9.2	6.7
4	Vegetable and bean hot pot	0.50	-0.072	-0.091	-0.053	8.5	6.5

4	Apple and kale juice[B]	0.52	-0.066	-0.082	-0.049	7.1	5.3
4	Ham and tomato muffin	0.43	-0.064	-0.084	-0.044	8.2	6.3
4	Caesar salad	0.43	-0.063	-0.082	-0.043	8.8	6.9
4	Vegetarian sausages	0.47	-0.062	-0.080	-0.045	8.6	5.1
4	Frozen yoghurt	0.47	-0.058	-0.074	-0.041	7.9	6.2
4	Raw snack vegetables	0.39	-0.057	-0.076	-0.038	8.0	6.2
4	Egg mayonnaise sandwich	0.30	-0.055	-0.078	-0.033	8.1	6.4
4	Mixed green salad	0.47	-0.055	-0.070	-0.039	8.6	7.0
4	Yoghurt	0.38	-0.055	-0.074	-0.037	8.8	7.1
4	Hot coffee[B]	0.23	-0.052	-0.078	-0.026	8.8	7.1
4	Camomile tea[B]	0.33	-0.051	-0.070	-0.031	6.6	5.2
4	Mixed raw nuts	0.29	-0.051	-0.072	-0.030	8.4	6.8
4	Iced coffee[B]	0.28	-0.050	-0.072	-0.029	7.4	6.0
4	Croissant	0.35	-0.049	-0.067	-0.031	8.6	7.1
4	Cheese and vegemite sandwich	0.17	-0.042	-0.067	-0.017	7.3	6.0
4	Scrambled eggs	0.18	-0.041	-0.065	-0.018	8.8	7.4
4	Sparkling water[B]	0.24	-0.037	-0.055	-0.019	6.8	5.7
4	Peanut butter sandwich	0.14	-0.035	-0.059	-0.011	7.4	6.4
4	Ham and cheese muffin	0.16	-0.033	-0.053	-0.012	7.6	6.6
4	Porridge/hot oatmeal	0.18	-0.033	-0.052	-0.015	7.2	6.2
4	Fresh fruit salad	0.33	-0.031	-0.042	-0.019	8.4	7.4
4	Instant noodles	0.18	-0.031	-0.048	-0.013	6.8	6.0
4	Lemon mousse tart	0.11	-0.028	-0.050	-0.006	7.2	6.4
4	Spaghetti Bolognese	0.15	-0.027	-0.045	-0.010	8.5	7.5
4	Banana	0.11	-0.026	-0.047	-0.006	7.9	7.1
4	Cereal/muesli	0.09	-0.023	-0.042	-0.003	7.5	6.9
4	Cold sliced meats	0.11	-0.022	-0.039	-0.005	7.6	6.8
4	Ham and cheese sandwich	0.07	-0.018	-0.036	-0.001	7.7	7.1
4	Fruit juice[B]	0.07	-0.014	-0.028	0.000	7.6	7.1
4	Water[B]	0.05 [ns]	-0.014	-0.030	0.002	8.3	7.8

4	Ham and cheese toasted sandwich	0.00 ns	-0.004	-0.020	0.012	7.5	7.2
4	Hash browns	0.00 ns	-0.004	-0.022	0.014	7.1	6.9
4	Sausage rolls	0.00 ns	0.000	-0.016	0.016	6.9	6.8
4	Buttered toast	0.00 ns	0.001	-0.012	0.015	7.2	7.2
4	Meat pie	0.01 ns	0.007	-0.009	0.023	6.6	6.7
4	Milkshake[B]	0.06 ns	0.012	-0.001	0.024	6.5	6.8
5	Thai green curry	0.88	-0.120	-0.132	-0.108	10.1	5.4
5	Sushi	0.76	-0.113	-0.129	-0.096	9.5	5.0
5	Prawn risotto	0.86	-0.104	-0.115	-0.093	9.3	5.2
5	Blue-vein cheese	0.83	-0.091	-0.102	-0.081	7.7	4.1
5	Spicy/hot chilli con carne	0.74	-0.090	-0.104	-0.076	8.4	4.9
5	Tuna steak	0.79	-0.086	-0.097	-0.074	8.4	5.0
5	Strong mustard	0.78	-0.084	-0.096	-0.072	8.0	4.7
5	Rabbit ragu	0.85	-0.082	-0.091	-0.072	6.9	3.7
5	Smoked cheese	0.77	-0.075	-0.086	-0.065	8.2	5.3
5	Pickled herring	0.74	-0.064	-0.074	-0.054	5.6	3.1
5	Brussel sprouts	0.48	-0.053	-0.068	-0.038	7.1	5.0
5	Dark chocolate	0.56	-0.040	-0.050	-0.031	8.1	6.5
5	Mild cheese	0.18	-0.026	-0.040	-0.011	7.7	6.7
5	Garlic bread	0.11	-0.020	-0.034	-0.005	8.3	7.5
5	White rice	0.18	-0.020	-0.031	-0.009	7.5	6.7
5	Apple	0.10	-0.011	-0.020	-0.003	7.6	7.2
5	Chicken breast	0.01 ns	-0.006	-0.022	0.009	7.7	7.5
5	White bread	0.20	0.018	0.009	0.028	6.0	6.7
6	Thai green chicken curry	0.91	-0.132	-0.143	-0.122	10.1	4.8
6	Chilli chicken stir-fry	0.86	-0.122	-0.135	-0.109	10.0	5.2
6	Chilli con carne	0.86	-0.112	-0.124	-0.100	9.8	5.4
6	Mild Indian curry (vegetarian)	0.83	-0.112	-0.126	-0.099	9.5	5.1
6	Chicken korma	0.76	-0.109	-0.125	-0.093	9.8	5.4
6	Spicy lamb meatballs	0.83	-0.106	-0.118	-0.093	9.1	4.9

6	Lamb kebabs	0.78	-0.099	-0.113	-0.086	9.3	5.4
6	Chickpea salad (spicy)	0.80	-0.095	-0.107	-0.082	7.9	4.2
6	Chicken fried rice	0.53	-0.076	-0.095	-0.057	9.3	6.3
6	Tuna pasta	0.54	-0.074	-0.092	-0.056	8.1	5.2
6	Soy milk[B]	0.72	-0.071	-0.083	-0.059	6.4	3.5
6	Sardines on toast	0.64	-0.070	-0.084	-0.056	6.6	3.8
6	Crackers with salmon pate	0.48	-0.068	-0.086	-0.049	7.1	4.4
6	Beef and beetroot sausages	0.67	-0.066	-0.078	-0.054	6.7	4.1
6	Mixed green salad	0.49	-0.065	-0.083	-0.048	8.5	5.9
6	Tuna steak	0.49	-0.065	-0.082	-0.047	8.0	5.5
6	Dairy-free yoghurt	0.51	-0.054	-0.068	-0.040	6.5	4.4
6	Savoury mince	0.35	-0.050	-0.068	-0.032	8.1	6.1
6	Pork and potato sausages	0.27	-0.036	-0.051	-0.020	6.9	5.4
6	Chicken casserole	0.25	-0.034	-0.050	-0.018	8.1	6.7
7	Chilli chicken stir-fry	0.78	-0.116	-0.134	-0.099	10.0	5.7
7	Pasta with sundried tomato and garlic meat sauce	0.64	-0.095	-0.115	-0.076	9.3	5.8
7	Wholemeal pasta salad with chicken	0.68	-0.094	-0.112	-0.076	8.5	5.0
7	Chicken and rice salad with spicy mayonnaise	0.71	-0.093	-0.109	-0.076	8.4	5.0
7	Spinach and artichoke pizza	0.66	-0.091	-0.109	-0.073	7.8	4.4
7	Salsa black bean burger	0.68	-0.087	-0.103	-0.071	6.7	3.5
7	Vegetarian pizza	0.59	-0.086	-0.105	-0.066	7.8	4.6
7	Stuffed bread with cheese and herbs	0.35	-0.068	-0.093	-0.042	8.6	6.1
7	Vegetarian meat loaf	0.52	-0.065	-0.082	-0.048	5.8	3.4
7	Ham and potato soup	0.34	-0.063	-0.087	-0.039	8.2	5.8
7	Chicken casserole	0.40	-0.058	-0.077	-0.038	8.3	6.2
7	Potato and lentil soup	0.35	-0.056	-0.077	-0.035	8.1	6.0
7	Spicy red cabbage	0.37	-0.056	-0.075	-0.036	7.2	5.2
7	Vegan bratwurst	0.32	-0.051	-0.072	-0.030	5.2	3.3
7	Bread and cheese	0.23	-0.047	-0.070	-0.023	8.8	7.1
7	Herring fillet in tomato sauce	0.22	-0.044	-0.066	-0.021	6.8	5.2

7	Spaghetti Bolognaise	0.17	-0.037	-0.059	-0.015	8.8	7.4
7	Meat loaf	0.08	-0.023	-0.044	-0.002	7.7	6.8
7	Sauerkraut	0.04 [ns]	-0.014	-0.034	0.006	6.9	6.3
7	Bratwurst	0.00 [ns]	-0.002	-0.018	0.013	7.2	7.1
8	Sushi	0.69	-0.121	-0.143	-0.100	9.4	4.7
8	Thai meal	0.73	-0.106	-0.123	-0.089	10.2	6.1
8	Kebab and green salad	0.49	-0.082	-0.104	-0.059	9.7	6.6
8	Toasted ryebread with fried egg and avocado	0.40	-0.081	-0.108	-0.055	9.5	6.3
8	Vegetarian pizza	0.53	-0.078	-0.098	-0.059	8.1	5.1
8	Pasta salad with feta cheese	0.39	-0.072	-0.096	-0.048	8.9	6.1
8	Roasted nuts	0.38	-0.072	-0.097	-0.048	9.4	6.6
8	Mixed nuts with dried fruits	0.37	-0.070	-0.094	-0.045	8.7	6.0
8	Spinach and tomato omelette	0.30	-0.068	-0.095	-0.040	9.4	6.8
8	Vegetable juice[B]	0.44	-0.067	-0.087	-0.047	7.4	4.9
8	Herbal tea[B]	0.42	-0.063	-0.083	-0.043	6.9	4.5
8	Cheese fondue	0.30	-0.061	-0.086	-0.036	7.1	4.8
8	Salmon and green salad	0.26	-0.058	-0.084	-0.032	8.8	6.6
8	Beer[B]	0.30	-0.054	-0.076	-0.032	7.7	5.6
8	Sparkling water[B]	0.21	-0.054	-0.082	-0.026	7.7	5.6
8	Mixed grilled vegetables	0.26	-0.052	-0.076	-0.028	9.4	7.4
8	Stewed apples	0.25	-0.052	-0.075	-0.028	7.8	5.8
8	Skyr with muesli	0.24	-0.050	-0.073	-0.026	7.0	5.1
8	Bun (Focaccia bread) with turkey, salad and dressing	0.29	-0.049	-0.069	-0.028	8.8	6.9
8	Ham and cheese quiche	0.26	-0.048	-0.070	-0.026	8.8	6.9
8	Quiche with leek and bacon	0.22	-0.043	-0.064	-0.021	8.7	7.1
8	Muesli with milk	0.17	-0.042	-0.067	-0.017	7.1	5.4
8	White bread roll with ham and cheese	0.15	-0.040	-0.066	-0.014	8.3	6.8
8	Fish cake on bread	0.18	-0.038	-0.059	-0.016	8.5	7.0
8	Wholemeal bread with jam	0.16	-0.038	-0.062	-0.014	7.9	6.4
8	Ryebread with sliced meat	0.13	-0.036	-0.062	-0.011	7.7	6.3

8	Ryebread with cheese	0.15	-0.033	-0.053	-0.012	8.2	7.0
8	Beef rissole and potato salad	0.16	-0.031	-0.049	-0.012	8.7	7.5
8	Raw vegetables: tomato, cucumber, cauliflower, capsicum	0.19	-0.030	-0.047	-0.014	8.7	7.5
8	Water[B]	0.13	-0.030	-0.051	-0.010	9.0	7.8
8	Coffee[B]	0.09	-0.026	-0.047	-0.004	7.7	6.7
8	Warm liver pate with mushrooms	0.12	-0.025	-0.043	-0.006	8.3	7.4
8	Fruit salad	0.10	-0.024	-0.043	-0.005	8.3	7.4
8	Porridge/Hot oatmeal	0.08	-0.024	-0.046	-0.003	6.5	5.6
8	Broth with vegetables and meat balls	0.11	-0.022	-0.038	-0.005	8.0	7.2
8	Soft boiled egg with bread	0.07	-0.020	-0.039	0.000	8.3	7.5
8	Banana	0.05 [ns]	-0.016	-0.034	0.002	8.2	7.5
8	Ryebread with mackerel in tomato sauce	0.04 [ns]	-0.016	-0.039	0.007	7.4	6.8
8	Milk and rice porridge	0.03 [ns]	-0.014	-0.035	0.006	7.3	6.7
8	Spaghetti Bolognese	0.08	-0.013	-0.025	-0.001	8.5	8.0
8	Orange juice[B]	0.01 [ns]	-0.005	-0.019	0.009	7.7	7.5
8	Danish pastry	0.00	-0.002	-0.024	0.020	6.8	6.7
8	French fries	0.01 [ns]	0.005	-0.012	0.021	7.4	7.6
8	Coca Cola[B]	0.06 [ns]	0.016	-0.001	0.033	6.0	6.6
8	French hotdog w/o dressing	0.09	0.017	0.002	0.032	6.3	7.0

Notes. *) B superscript indicates that the stimulus is a beverage. For Studies 7 and 8, refer to Part 2 of Supplementary Material for the stimuli wordings in original languages.

Figure S1. Distribution of summed FN scores for Study 1 to show that the number of observations underpinning each FN value was highly variable since there are more participants with FN scores in the mid-range than at the extremes of the scale range. This is particularly true for participants with high levels of FN.

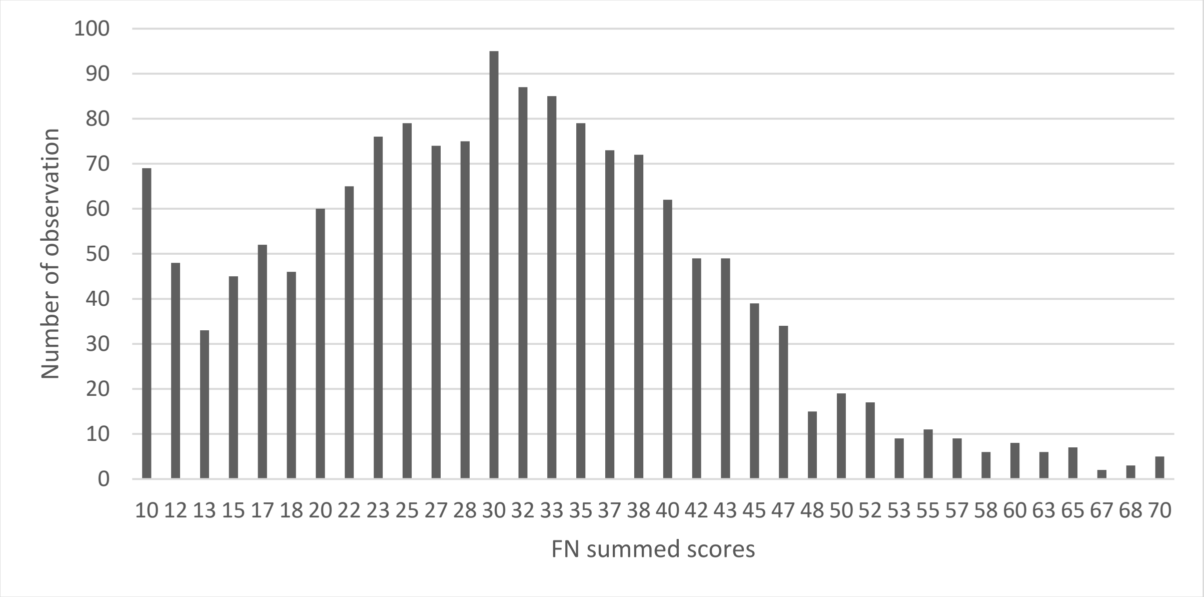

Table S4. Overview of the six groups of F&B names with variable impact of FN on liking. The number of F&B names in each group is given together with summary statistics for the regression coefficients that were input to the discretization procedure. The names given to each group are qualitative labels to express differences in the direction and strength of the relative relationship between FN and liking.

**Group name for effect of FN on liking**	**Nbr. F&B names**	**Reg. Coeff. Mean (HSD)**	**Reg. Coeff. 95% Conf. Interval**	
Neg. – Very High	18	-0.114 [F]	-0.132	-0.099
Neg. – High	54	-0.082 [E]	-0.097	-0.071
Neg. – Medium	64	-0.058 [D]	-0.070	-0.048
Neg. – Low	34	-0.038 [C]	-0.047	-0.030
Neg. – Very Low	27	-0.020 [B]	-0.028	-0.002
Pos. – Very Low	2	0.018 [A]	0.017	0.018

Notes. Neg = negative, Pos = positive. The mean values for regression coefficients are shown with results from Tukey's HSD, where groups which share a letter are not significantly different at the 5% level.

Figure S2. Illustration of the variable effect of average F&B liking as a function of FN: a) a significant and weak positive linkage between FN and liking - 'white bread' (AU), b) a significant and weak negative linkage between FN and liking from the "very low" group - 'blueberry muffin' (US), and c) a significant and strong negative linkage between FN and liking from the "high" group - 'vegetarian pizza' (DE). F&B liking measured on 9-pt scale from 1= 'dislike extremely' to 9='like extremely.' Theoretical range for FN was 10 to 70, where higher values correspond to higher levels of FN. The number of respondents contributing to each point vary as dictated by the shape of the underlying FN distributions.

(A)

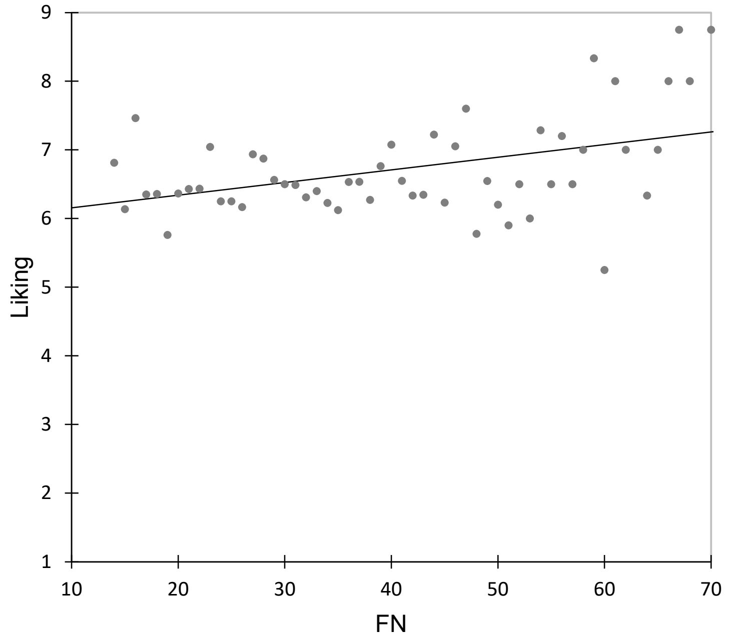

(B)

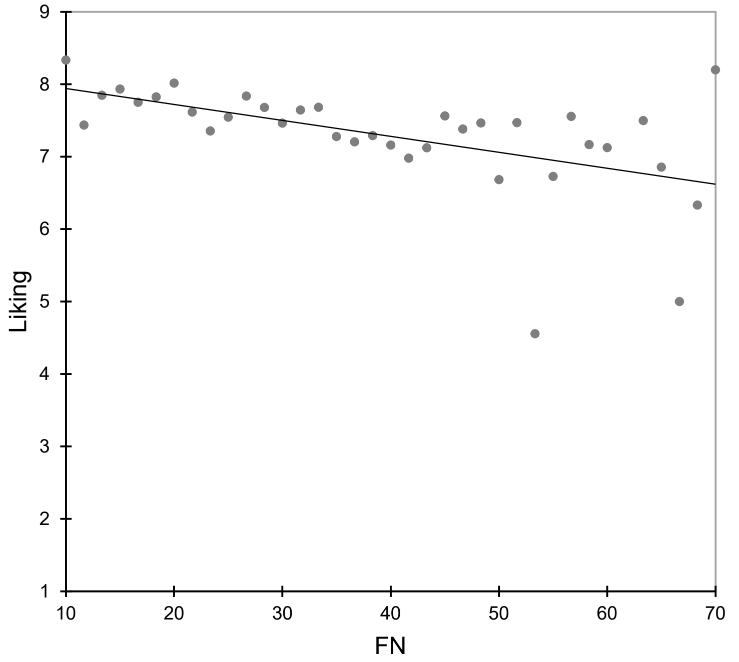

(C)

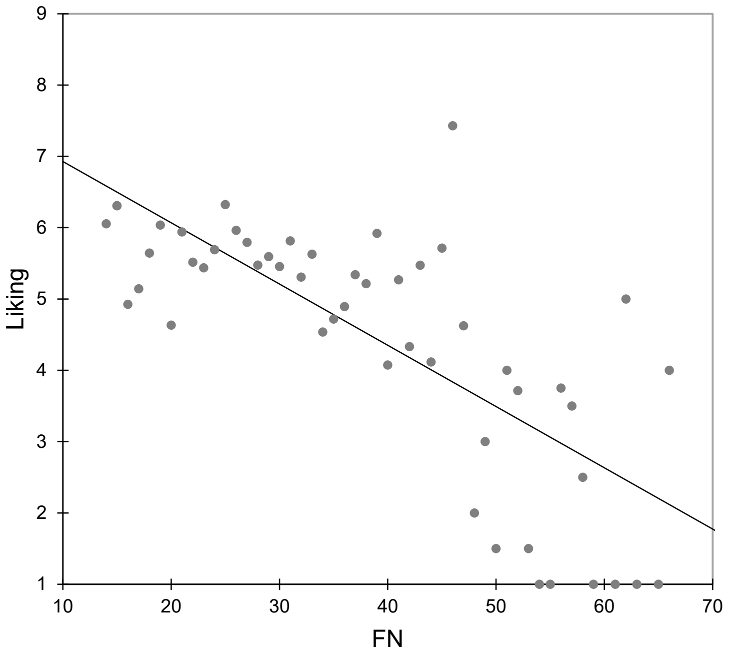

Supplement: Supplementary file 1 [file nutrients-13-03657-s001.zip › nutrients-1407276-supplementary.pdf]
